# A Dipeptide Module Substitution-Based Strategy for the Modification and Evaluation of ACE Inhibitory Peptides

**DOI:** 10.3390/foods15203617

**Published:** 2026-10-11

**Authors:** Qingping Liang, Shuangtong Wang, Nana Sun, Changliang Zhu, Zhemin Liu, Haijin Mou

**Affiliations:** 1 College of Food Science and Engineering, Ocean University of China, Qingdao 266404, China; preenyliang@126.com (Q.L.);; 2 School of Ocean Food and Biological Engineering, Jiangsu Ocean University, Lianyungang 222005, China; 3 Jiangsu Key Laboratory of Marine Bioresources and Environment/Jiangsu Key Laboratory of Marine Biotechnology, Jiangsu Ocean University, Lianyungang 222005, China

**Keywords:** ACE inhibitory peptide, rational design strategy, dipeptide module substitution, structure–activity relationship

## Abstract

Food-derived angiotensin-converting enzyme (ACE) inhibitory peptide LYPVK exhibits promising potential for application as an important ingredient with a potential antihypertensive effect in functional foods. However, the structure–activity relationship and the rational design strategy for peptide modification remain insufficiently understood. To address this, dipeptide module substitution was employed to modify and investigate the mechanism of this food-derived ACE inhibitory peptide, providing an insight into the structure–activity relationship. Through integration of molecular docking and activity verification, LY and YP within LYPVK were identified as potential low- and high-activity fragments, respectively. Meanwhile, EY and SY were screened as high-contribution ones, resulting in the generation of EYPVK and SYPVK, which exhibited 13.5% and 25.1% higher ACE inhibitory activity, respectively, compared with the original peptide LYPVK. The enhanced activity may be attributed to increased hydrogen bonds and hydrophobic interactions with ACE and the strengthening of Tyr-mediated binding by Glu or Ser residues in the peptide sequences. Cellular assays showed altered nitric oxide (NO) and endothelin-1 (ET-1) levels, suggesting their potential function at the endothelial cell level. Additionally, SYPVK may regulate multiple targets and pathways for its function, as indicated by the network pharmacology and real-time fluorescence quantitative PCR analysis. These findings demonstrate that dipeptide module substitution is a viable approach for peptide optimization, highlighting the potential module effects in the design of functional peptides.

## 1. Introduction

Hypertension remains a major global public health concern that substantially elevates the risk of cardiovascular disease, stroke and renal failure [1]. Angiotensin I-converting enzyme (ACE) plays a crucial role in the regulation of blood pressure through two primary mechanisms, including the conversion of angiotensin I into the vasoconstrictive peptide angiotensin II and the degradation of the vasodilatory peptide bradykinin [2]. Accordingly, inhibition of ACE has emerged as a well-established therapeutic strategy for the management of hypertension [3]. In recent years, bioactive peptides with ACE inhibitory activity have drawn growing attention as safer, more natural alternatives to synthetic pharmaceuticals, especially in the fields of functional foods and nutraceuticals, due to the side effects associated with long-term drug administration [4].

ACE inhibitory peptides generally consist of 2 to 12 amino acids and demonstrate activity that is strongly dependent on their sequence amino acid composition and arrangement [5]. Previous studies have identified several structural features that contribute to their potency, including the presence of hydrophobic residues, Pro or aromatic amino acids at the C-terminus, hydrophobicity, and the net charge distribution [6]. Despite the identification of numerous ACE inhibitory peptides from food protein sources, the mechanisms underlying their structure–activity relationships remain incompletely elucidated. Additionally, naturally extracting peptides often have limitations that constrain their practical applications, including suboptimal bioactivity, poor bioavailability, and a short biological half-life, which are generally improved currently using some rational design strategy [7]. The most widely adopted rational design strategy in peptide design is sequence optimization. Nevertheless, the current advancement of strategies is inadequate to further improve the performance for the practical applications of functional peptides, enabling the development of more efficient ration design strategies necessary.

In recent years, the concept of modules has been introduced into peptide engineering to facilitate rational modification to improve peptide activity and structure–activity relationship analysis. In the areas of peptide engineering and design, a module can be defined as a sequence unit composed of consecutive amino acid residues. Such modules are not necessarily structurally independent, which are evaluated as a single entity during sequence analysis. Depending on the peptide length and design objective, modules may consist of dipeptides, tripeptides, or longer sequence segments. In contrast to conventional single-residue substitution, module-based design allows for the collective properties and interactions of associated amino acid residues in a local sequence region to be explicitly accounted for during peptide optimization. Previous studies have demonstrated the feasibility of module substitution for improving peptide functionality [8,9]. Nevertheless, the biological activity of an isolated module cannot directly serve as a measure of its contribution within the peptide sequence because its functional effect may depend on sequence composition, associated residues, and terminal position. Therefore, it can serve as an approach to identify candidate sequence regions and substitution modules, whereas the effectiveness of the proposed modification requires subsequent validation in the full-length peptide.

In the present study, we aim to improve the ACE inhibitory activity of the original peptide LYPVK, which was studied in our previous study, through a dipeptide module substitution design strategy. Combining molecular docking and activity verification approaches, dipeptide modules within the LYPVK sequence with different activity were divided and identified. The low-activity component was replaced by dipeptide modules with higher activity identified from various dipeptide arrangements, resulting in the generation of two novel peptides. Their ACE inhibitory activity was verified and compared with that of the original peptide, which verified the efficacy of the module substitution strategy for improving the inhibitory activity of peptides. Gastrointestinal digestion stability and the ACE inhibition pattern were subsequently evaluated. Molecular docking and molecular dynamics (MD) simulations were employed to investigate the binding interactions between these peptides and ACE, aiming to elucidate the underlying mechanisms responsible for changes in activity and properties from the perspectives of binding affinity and intermolecular forces brought about by module substitution. Additionally, the functions of these peptides on endothelial cells were investigated in human umbilical vein cell fusion cells (EA.hy926). Network pharmacology analysis was employed to identify potential action targets and elucidate the underlying mechanisms of the peptide, with the most potent ACE inhibitory activity obtained after module substitution. The novelty of this study lies in the application of this strategy for LYPVK, which, adopted in this study, aims to improve peptide functionality while advancing the understanding of sequence–activity relationships for ACE inhibition.

## 2. Materials and Methods

### 2.1. Materials and Reagents

ACE, 2-[4-(2-hydroxyethyl) piperazin-1-yl] ethanesulfonic acid (HEPES) and N-[3-(2-furylacryloyl)]-L-phenyalanyl-glycyl-glycine (FAPGG) were purchased from Sigma-Aldrich (St. Louis, MO, USA). Dulbecco’s modified Eagle’s medium (DMEM), 1% penicillin/streptomycin (P/S), trypsin-EDTA (0.25%), and fetal bovine serum (FBS) used for cell culture were provided by Beijing Suolaibao Technology Co., Ltd. (Beijing, China). Cell counting kit-8 (CCK-8), nitric oxide (NO) and endothelin-1 (ET-1) assay kits were purchased from Nanjing Jiancheng Bioengineering Co., Ltd. (Nanjing, China). The peptides used in this study were synthesized by the Shanghai Jier Biological Technology Co., Ltd. (Shanghai, China) with a purity of more than 95%. All other chemicals and reagents used were of analytical grade.

### 2.2. Module Activity Confirmation of Dipeptides Within LYPVK

#### 2.2.1. Dipeptide Modules Division and Molecular Docking

The LYPVK sequence was divided into four dipeptide modules, including LY, YP, PV, and VK, which served as units for module-level screening, rather than being treated as structurally independent peptides embedded in the parent peptide sequence. These modules were subsequently analyzed using molecular docking to assess the activity of dipeptides. Specifically, the three-dimensional (3D) structures of each dipeptide module with the minimal energy were constructed by Discovery Studio 2019 software, and each peptide was optimized under the MM2 force field to minimize its energy. The 3D structure of the ACE was retrieved from the Protein Data Bank (PDB) database with the PDB ID of 1o86, and water and ligands from the receptor protein and added hydrogen were removed. The molecular docking procedures between each dipeptide module and ACE were carried out using the CDOCKER model, with the zinc-centered cavity located at coordinates x = 40.997, y = 34.127, and z = 46.348 (radius: 12 Å). The molecular docking results, such as the optimal binding pose with the lowest binding energy, two-dimensional (2D) interactions and key binding residues, were further analyzed using Discovery Studio 2019.

#### 2.2.2. ACE Inhibitory Activity Assay

The in vitro activity of the dipeptide module within LYPVK was subsequently evaluated using the 96-well plate method, as previously described, with minor modifications [10]. Briefly, 50 µL of 1 mM FAPGG served as the substrate, and 10 µL of 0.1 U/mL ACE was added along with 40 µL of the tested samples at various concentrations in a 96-well plate. For the blank control, 40 µL of HEPES buffer without any sample was added to the reaction system in the same plate format. The reaction system was prepared, and the absorbance was measured at 340 nm. Subsequently, the absorbance measurement at 340 nm was conducted after incubation at 37 °C for 30 min, and the difference between pre- and post-reaction absorbance values was defined as the change in absorbance for both the sample and blank groups. The ACE inhibitory rate (%) was calculated using the formula: (1-change in absorbance for the sample group/change in absorbance for the blank group) × 100%. IC_50_ values of the tested samples were defined as the concentrations at which the ACE inhibition rate reached 50%. The concentration–response data were fitted by nonlinear regression using a four-parameter logistic model in Origin 2024, and IC_50_ values were obtained from the fitted curves.

### 2.3. Screening of Candidate Dipeptide Modules for Substitution

The potential ACE inhibitory activity of 400 possible dipeptides was initially screened by molecular docking, which was used to evaluate their predicted interactions with ACE. Based on the results obtained from the module-level analysis of LYPVK, selected “XY” dipeptides were subsequently evaluated for their in vitro ACE inhibitory activity. The ACE inhibitory activity was measured following the procedure described in Section 2.2.2. The combined molecular docking and experimental activity results were used to identify candidate dipeptide modules for subsequent substitution. The relationship between molecular docking interaction energy and experimentally determined IC_50_ values was assessed using a two-sided Pearson correlation test. Each “XY” dipeptide contributed one paired observation. A simple linear regression line and its 95% confidence interval were plotted to visualize the relationship.

### 2.4. Module Substitution and Full-Length Peptide Activity Validation

Based on the analysis of the activity of dipeptide modules, the lower activity modules within the LYPVK sequence were replaced with the identified high-activity dipeptide modules. The resulting full-length peptides were subsequently evaluated for ACE inhibitory activity and compared with the parent peptide LYPVK to determine whether the proposed module substitution strategy enhanced peptide activity. The ACE inhibitory activity was measured following the procedure described in Section 2.2.2.

### 2.5. Gastrointestinal Digestion Resistance Assay

The gastrointestinal digestion resistance of ACE inhibitory peptides was evaluated in vitro under simulated gastrointestinal digestive conditions [11]. Each peptide was subjected to digestion at its IC_50_ concentration. This concentration was selected to provide an initial ACE inhibitory level of approximately 50% for each peptide, thereby allowing the change in inhibitory activity during digestion to be evaluated relative to the activity after digestion. The resulting inhibition rates were used to assess the residual activity within each peptide for understanding their resistance to various environments. Accordingly, the tested peptide sample at a concentration of its IC_50_ value was adjusted to pH 2.0 using 1 M HCl, and pepsin (3500 U/g) was added at an enzyme-to-substrate ratio of 2% (*w*/*w*) for a duration of 2 h at 37 °C, with sample collection performed every 30 min throughout the digestion process. The remaining samples were treated with 2% trypsin (E/S, *w*/*w*, 1250 U/g) at pH 7.5 for an additional 3 h. During this period, 50 μL of the enzymatic digestion product was collected every 30 min and subsequently inactivated by boiling at 95 °C for 10 min to determine the in vitro ACE inhibition rate. The method used to detect the ACE inhibition rate follows the procedure described in Section 2.2.2.

### 2.6. Inhibitory Kinetics Study

The Lineweaver–Burk plot was performed to determine the inhibition pattern of peptides against ACE. Each peptide sample at varying concentrations (0, 50, and 100 μg/mL) was introduced into the reaction system containing the substrate FAPGG at different concentrations (0.5, 1.0, and 2.0 mM). The Lineweaver–Burk plot was employed to determine the maximum reaction rate (V_max_) and the Michaelis–Menten constant (K_m_), in order to analyze the inhibitory pattern of the peptides against ACE.

### 2.7. Molecular Docking and MD Simulation Analysis

The molecular docking between the peptides generated after substitution and the active sites of ACE was conducted to further elucidate the intermolecular interactions and binding sites, thereby investigating the potential mechanism underlying the alteration of biological activity, as well as the potential module effects. The molecular docking procedure was consistent with the methods described in Section 2.2.1. Subsequently, MD simulations of the peptide–ACE binding complexes were conducted using Gromacs 2022.3 software to assess their structural stability. RESP atomic charges for the peptides were derived from quantum-chemical calculations using Gaussian 16W, and peptide parameters were generated with AmberTools 22 using the GAFF force field. ACE was described using the AMBER ff99SB-ILDN force field. Each peptide–ACE complex was placed at the center of a periodic cubic box (10 × 10 × 10 nm^3^) and solvated explicitly with TIP3P water, maintaining a minimum solute-to-box boundary distance of 1.0 nm. Initial structural relaxation was performed by steepest-descent energy minimization for a maximum of 50,000 steps, followed by conjugate-gradient minimization. The minimized systems were gradually heated from 0 to 300 K over 100 ps under the NVT ensemble using the V-rescale thermostat. Subsequently, the systems were equilibrated for 1 ns under the NPT ensemble at 300 K and 1 bar, with pressure maintained using the Parrinello–Rahman barostat. Production simulations were subsequently performed for 100 ns under periodic boundary conditions at 300 K and 1 bar using a 2 fs integration timestep. Long-range electrostatic interactions were calculated using the particle mesh Ewald (PME) method, while hydrogen bonds were constrained using the LINCS algorithm. The post-simulation analysis included trajectory analysis, utilizing indicators such as root mean square deviation (RMSD), root mean square fluctuation (RMSF), the number of hydrogen bonds, and solvent-accessible surface area (SASA) to evaluate the stability of the complexes.

### 2.8. Evaluation of Antihypertensive Effects on Endothelial Cells

#### 2.8.1. Cell Culture

The EA.hy926 cell line was procured from Suzhou Haixing Biosciences Co., Ltd. (Suzhou, China), which is a human umbilical vein fusion cell line. This hybrid cell line, originally established by fusing human umbilical vein endothelial cells (HUVECs) with A549 cells, is a well-validated model for endothelial function studies. The identity and purity of the cell line were authenticated by the supplier using short tandem repeat (STR) profiling, with the genotyping results demonstrated in our previous study [12]. The cells were cultured in DMEM supplemented with 10% FBS and 1% P/S at 37 °C under a 5% CO_2_ atmosphere. The culture medium was renewed every 2–3 days until the cells in the culture flask reached approximately 90% confluence, after which the cells were subcultured using 0.25% trypsin-EDTA. The cells were used to conduct the experiments after three to five passages.

#### 2.8.2. Cell Proliferation Assay by CCK-8

The cells were diluted to a density of 2.0 × 10^4^ cells per well in a 96-well plate and subsequently cultured to allow for adherent growth. Following a 24 h starvation period using DMEM basal culture medium, the peptides were diluted to various concentrations (0, 2.5, 5, 10, 20, and 40 µg/mL) with the same medium and subsequently added to the cells in a 96-well plate for a 24 h incubation. Cell viability was then evaluated by employing the CCK-8 assay kit according to the instructions.

#### 2.8.3. No Production and ET-1 Secretion Content Determination

EA.hy926 cells were seeded in 24-well plates at a density of 1 × 10^5^ cells per well and incubated for 24 h. Peptide samples at different concentrations (0, 10, and 20 µg/mL) were added to incubate the cells for another 24 and 48 h. Captopril at 10 µg/mL served as a positive control. The cell supernatants were collected to measure the NO production and ET-1 contents in accordance with the provided guidelines of the assay kit. The NO and ET-1 contents were measured using commercially available assay kits purchased from the Nanjing Jiancheng Bioengineering Institute (Nanjing, China).

### 2.9. Potential Hypertension-Related Targets Mining of SYPVK

Potential targets associated with SYPVK were predicted using the PharmMapper database (http://lilab-ecust.cn/pharmmapper/index.html, accessed on 5 September 2025) to establish the potential active target sets of SYPVK [13]. The gene names of the predicted targets were standardized using the UniProt database, and the hypertension-associated gene set was constructed based on the GeneCards database (https://www.genecards.org/). The gene set was retrieved using the search term “hypertension”, with filters restricting the species to Homo sapiens and retaining only genes with a relevance score greater than 2. The intersection between the predicted SYPVK-associated targets and hypertension-associated targets was determined, resulting in the overlapping candidate targets, which were identified by STRING database. These genes were then analyzed using Cytoscape 3.9.1 software to construct a protein–protein interaction (PPI) network, which facilitated the identification of potential therapeutic targets of SYPVK.

### 2.10. GO Bio-Functional Analysis and KEGG Pathway Enrichment Analysis

The DAVID database was employed to gather data for Gene Ontology (GO) and Kyoto Encyclopedia of Genes and Genomes (KEGG) pathway enrichment analysis [14], which were conducted to explore the biological functions associated with the potential targets of SYPVK. GO analysis reflects the main biological functions of the potential targets, including biological process (BP), cellular component (CC), and molecular function (MF) [15]. KEGG pathway enrichment analysis was conducted using an FDR threshold of less than 0.05 to identify the primary pathways.

### 2.11. Molecular Docking for SYPVK and Core Targets

The molecular docking of the SYPVK to core targets was employed to further understand the intermolecular interactions between them. The 3D structures of the core targets AKT1 (PDB ID: 7NH4), ACE (PDB ID: 1o86), proto-oncogene tyrosine-protein kinase SRC (SRC) (PDB ID: 1a07), renin (REN) (PDB ID: 2V0Z), and albumin (ALB) (PDB ID: 1ao6) were obtained from the PDB database. Each receptor structure was prepared independently by removing crystallographic water molecules and nonessential heteroatoms, followed by the addition of hydrogen atoms. Functionally relevant cofactors were retained where appropriate, including the catalytic Zn^2+^ ion of ACE. Target-specific docking regions were defined independently according to the corresponding experimentally characterized or predicted ligand-binding pockets. The coordinate positions of the docking runs were x = 2.505, y = 3.995, z = 10.467 for AKT1 (7NH4); x = 40.124, y = 37.205, z = 43.371 for ACE (1O86); x = 24.8, y = 16.5, z = 28.7 for SRC (1A07); x = 34.136, y = 20.712, z = 83.300 for REN (2V0Z); and x = 31.6, y = 37.2, z = 28.4 for ALB (1AO6). The molecular docking between peptide and these core potential targets was performed using AutoDock Vina 1.5.7 software, and the docking results were analyzed using Discovery Studio 2019.

### 2.12. Quantitative mRNA Expression Analysis of Potential Targets in Cells

EA.hy926 were seeded in 6-well plates at a density of 1 × 10^5^ cells per well and incubated for 24 h, which were subsequently treated with SYPVK and the positive control captopril. Total RNA was extracted with the RNA extraction kit (Qingdao Mdbio Biotech Co., Ltd., Qingdao, China), and it was reverse transcribed using the Prime ScriptTM PT reagent kit (Takara, Shiga, Japan) to acquire the cDNA of each sample. The gene expression of potential targets was analyzed by real-time fluorescence quantitative PCR (RT-qPCR) using SYBR Green fluorescence on an RT-qPCR System (JASCO, Tokyo, Japan). The relative mRNA expressions of the tested genes were determined through normalization with respect to the levels of the control gene GAPDH, and the primers used for the tested genes are listed in Table S1.

### 2.13. Statistical Analysis

All experiments were performed in three independent experimental replicates (*n* = 3), and data are presented as the mean ± standard deviation (SD). Statistical analysis was conducted using IBM SPSS Statistics 26 software. For experiments involving one independent factor with more than two groups, one-way analysis of variance (ANOVA) followed by Tukey’s post hoc multiple-comparison test was used. For experiments involving both peptide concentration and incubation time, two-way ANOVA was used to evaluate the main effects of concentration and time by the Bonferroni adjustment for multiple comparisons. Statistical significance was defined as *p* < 0.05.

## 3. Results and Discussion

### 3.1. Activity Confirmation of Dipeptide Modules Within LYPVK

The ACE inhibitory peptide LYPVK, derived from *Ficus carica*, which demonstrates potent ACE inhibitory activity with an IC_50_ value of 17.1 ± 1.4 μM, was previously reported in our study [16]. Based on the potential molecular structure–activity relationship of ACE inhibitory peptides, novel peptides with enhanced inhibitory activity were designed and successfully investigated, thereby demonstrating the effectiveness of a sequence-based rational design strategy [12]. To develop a greater number of efficient and rational design strategies for the development of potent ACE inhibitory peptides, LYPVK was selected as the template sequence in this study to undergo sequence optimization through a module substitution approach.

Amino acids are the fundamental components of bioactive peptides, while the biological activity of a peptide is determined not only by individual residues but also by their combinations and neighboring interactions [17,18,19]. From the perspective of sequence-based rational design, sequence fragments containing two or more consecutive residues can therefore be treated as the modules for screening and substitution. Depending on the design objective, such modules may consist of dipeptides, tripeptides, or longer sequence fragments during the sequence analysis and strategy utilization. In the present study, dipeptides were selected as the minimum module unit because they incorporate information from neighboring residue combinations while maintaining the simple structure for systematic screening. Accordingly, LYPVK was divided into the four dipeptide modules—LY, YP, PV, and VK—for preliminary module-level analysis. Importantly, these modules were employed as sequence-design units rather than being assumed to represent structurally independent functional peptides within LYPVK.

**Figure 1:**
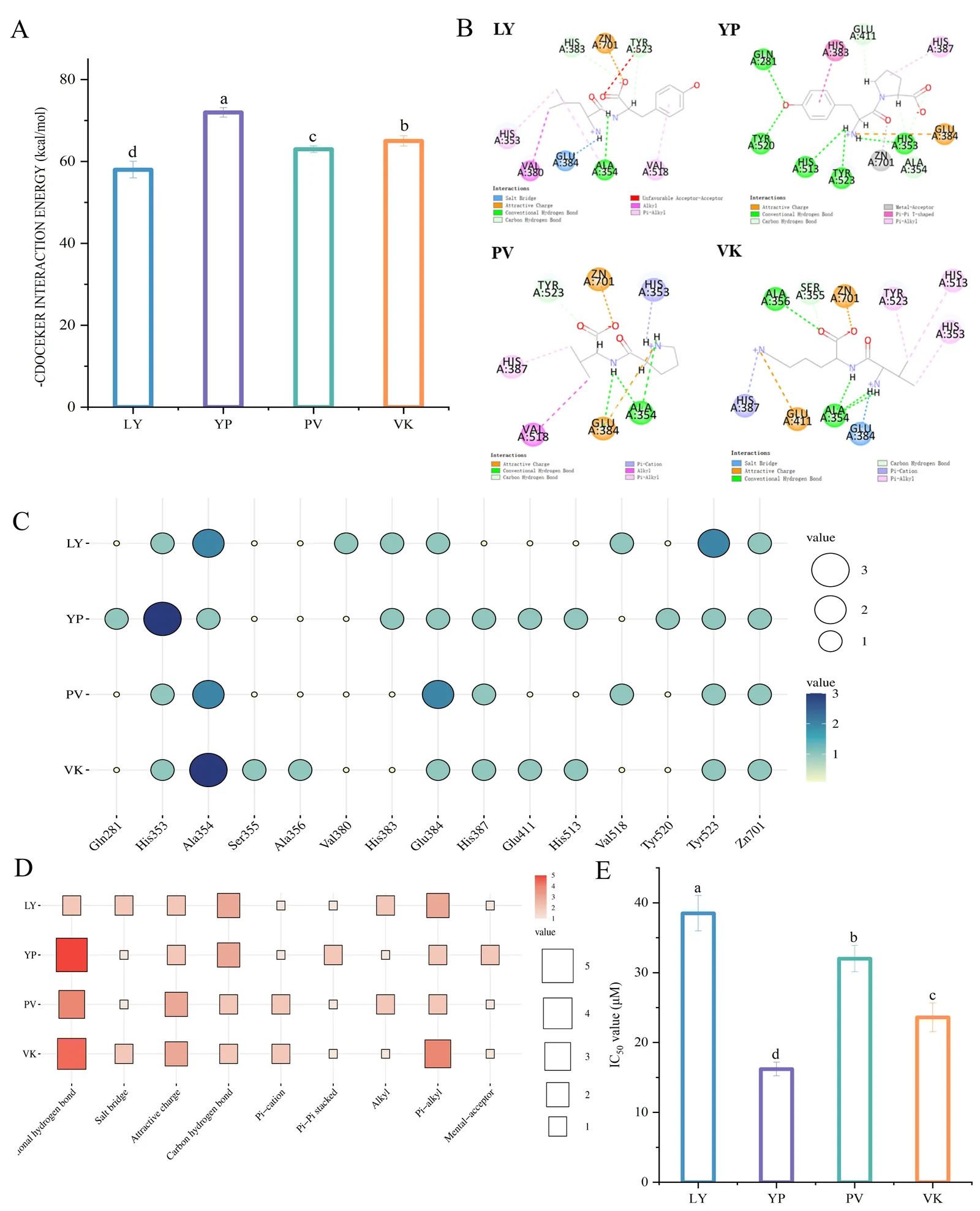
The prediction and verification of contribution of dipeptide modules in LYPVK sequences for ACE inhibitory activity. (**A**): Molecular docking scores of each dipeptide in LYPVK and ACE. (**B**): Molecular docking analysis of 2D detailed diagram of the interactions between each dipeptide in LYPVK and ACE. (**C**): Interaction residue diagrams between each dipeptide and ACE. (**D**): Interaction type diagrams between each dipeptide and ACE. (**E**): IC_50_ values of each dipeptide modules against ACE. Data were presented as mean ± SD (*n* = 3). ^a–d^ Significant differences among all different peptide samples at the same time were obtained using one-way ANOVA and indicated with different letters (*p* < 0.05).

Molecular docking was first employed to compare the predicted interactions of the four isolated dipeptide modules with ACE. The calculated interaction energies for LY, YP, PV, and VK were −58.286, −72.736, −63.269, and −65.168 kcal/mol, respectively (Figure 1A), with YP showing the most favorable predicted interaction and LY the least favorable among the four tested dipeptides. This indicates that the binding affinities of the four dipeptides for ACE, in descending order, are YP, PV, VK, and LY, owing to generally the lower docking energy values corresponding to stronger binding capacities [20]. Analysis of the interaction patterns further revealed differences in hydrogen bonding, interaction residues, and engagement with residues located within the ACE active-site pockets (Figure 1B–D). Specifically, molecular docking also played a crucial role in elucidating the action mechanism of ACE inhibitory peptides, including interaction types and active binding sites [21]. YP formed the highest number of hydrogen bonds with ACE, followed by VK, PV, and LY, indicating the order of their binding affinity, which is based on the widely accepted notion that hydrogen bonds represent the primary mode of interaction between ACE and peptides [21,22]. The active site is also a key factor that influences the activity of the peptide against ACE [23]. YP and VK interacted with the highest number of active sites within ACE, indicating their strong binding affinity and thereby suggesting their potential to exert bioactive effects (Figure 1D). Although a comparable number of interaction sites were observed between LY and PV, the greater number of binding sites within the ACE active center, such as Glu384, His387, and Ala354, contributed to the stronger interaction between PV and ACE (Figure 1C).

Meanwhile, the in vitro ACE inhibitory activity of the four dipeptide modules was evaluated to further elucidate the function of each dipeptide module within the LYPVK sequence. The inhibitory activity of each dipeptide sample at various concentrations was measured to determine their IC_50_ values against ACE. It was demonstrated that LY, YP, PV, and VK exhibited IC_50_ values of 38.50 ± 2.53, 16.24 ± 0.98, 32.15 ± 1.89, and 23.68 ± 2.06 μM, respectively (Figure 1E). These results were used for module-level screening of each dipeptide within LYPVK, and the relatively weak activity and less favorable predicted ACE interaction of LY suggested the N-terminal LY module as a candidate region for subsequent sequence optimization. Actually, the inhibitory activities of isolated dipeptides were used as the screening dipeptides for selecting candidate fragments for substitution. These activities do not directly quantify the contributions of the corresponding fragments within LYPVK, where associated residues, terminal position, and peptide conformation may influence ACE binding. LY and YP were served as fragments showing relatively low and high activities, respectively.

### 3.2. Screening of Candidate Dipeptide Modules for Substitution

**Figure 2:**
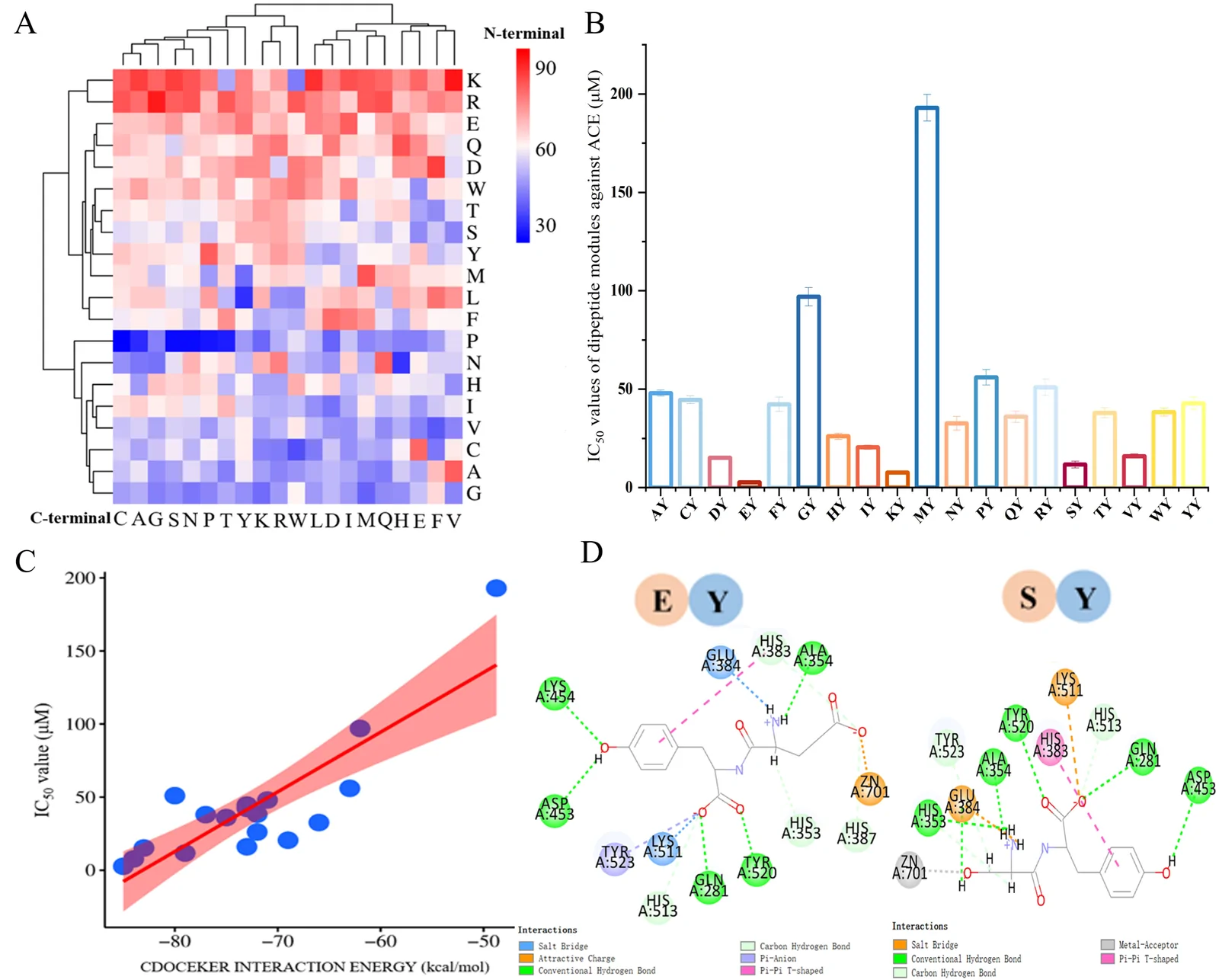
The prediction and verification of contribution of “XY” dipeptide modules for ACE inhibitory activity. (**A**): Molecular docking scores of each “XY” dipeptide and ACE. (**B**): IC_50_ values. (**C**): Correlation analysis between the molecular docking score and activity of “XY” dipeptide modules. Correlation between molecular docking interaction energy and experimentally determined IC_50_ values of the “XY” dipeptides. Each point represents one dipeptide (*n* = 19). The red line represents the linear regression fit, and the shaded region indicates its 95% confidence interval. Pearson’s *r* = 0.84; two-sided *p* = 7.6 × 10^−6^. (**D**): Molecular docking analysis of 2D detailed diagram of the interactions between screened high-contribution dipeptide (EY and SY) and ACE.

Based on the activities of the isolated dipeptides LY and YP, we used the “XY” sequence to screen candidate dipeptides for substitution. Molecular docking analysis predicted the potential ACE inhibitory activity of 400 dipeptide modules by quantifying the interaction energy between ACE and each individual dipeptide (Figure 2A). It was observed that EY, SY, KY, and VY showed the highest ACE inhibition activity, with an IC_50_ value of 2.68 ± 0.05, 11.70 ± 1.75, 7.70 ± 0.86, and 16.12 ± 1.04 μM, respectively, whereas GY and MY showed the lowest activity against with high IC_50_ values (Figure 2B). The measured activity results were consistent with the findings of a previous study, which indicated that charged and hydrophobic amino acids contribute to ACE inhibitory activity [6]. Docking interaction energy was associated with the experimentally determined IC_50_ values of the tested “XY” dipeptides (Pearson’s r = 0.84, two-sided *p* = 7.6 × 10^−6^, Figure 2C). However, individual dipeptides deviated from the fitted relationship, indicating that docking energy alone did not fully account for their ACE inhibitory activities. Therefore, docking was used as a complementary criterion for candidate prioritization, while the experimentally measured IC_50_ values remained the primary measure of inhibitory activity. Although several studies have employed molecular docking in conjunction with activity verification methods to predict the functional diversity of peptides with varying chain lengths, ranging from two amino acids to eight or more, this approach is generally more appropriate for small molecule interactions and less suitable for peptide molecules [8,24,25]. Considering this, the dipeptide ligand was selected, as the module may contribute to the high prediction accuracy of the “XY” dipeptide module.

The selection of modules for subsequent substitution was based on a combined assessment of the experimentally determined ACE inhibitory activity and molecular docking results. Experimental ACE inhibitory activity was used to identify the relatively active dipeptides. EY was selected because it had the lowest measured IC_50_ (2.68 ± 0.05 μM). SY had the lowest predicted interaction energy among, and its predicted pose showed significantly more numbers of hydrogen bonds relative to KY (Figure S1). Although KY had a lower measured IC_50_ than SY (7.70 ± 0.86 and 11.70 ± 1.75 μM), these docking observations led us to select SY as a second candidate for substitution. Therefore, based on the combined evaluation of in vitro ACE inhibitory activity, docking energy, and predicted interaction patterns, EY and SY were selected as candidate substitution modules within the template module sequence “XY”, followed by a detailed analysis of their interactions with ACE. It is important to emphasize that hydrogen bonds and hydrophobic interactions play a critical role in stabilizing peptide structures and facilitating their binding to the active sites of ACE. It was observed that EY and SY formed many hydrogen bonds with ACE, respectively, whereas the original LY module formed only one hydrogen bond, indicating varying levels of activity among these modules (Figure 2D). Additionally, although the EY and SY modules were observed to interact with multiple key residues within the active pockets, albeit to varying degrees and with differing interaction strengths, the total number of active sites involved in these interactions exceeds that of the original LY module (Figure 2D). Notably, SY forms a metal–acceptor interaction with the Zn^2+^ ion at the active site, which enhances the binding affinity of the SY module for the ACE active site and may contribute to its strong ACE inhibitory activity (Figure 2D).

### 3.3. Module Substitution Strategy Improved the Activity of Full-Length Ace Inhibitory Peptide

Modification of bioactive peptide using the efficient strategy can significantly enhance functional properties, thereby serving as a crucial strategy for improving practical applicability [8]. Currently, commonly employed peptide modification strategies primarily involve the rational design of peptide sequences based on the properties of amino acid residues and factors influencing biological activity, as well as computational tools for de novo design and chemical modifications of peptides. The rational design of peptide sequences relies on the identification and application of underlying sequence patterns that are associated with specific peptide functions [12]. Computer-aided de novo design approach enables the generation of novel functional peptides through computational simulation, while it entails substantial computational costs and is constrained by relatively low success rates [26]. Chemical modification involves higher synthesis costs owing to accurate process such as cyclization [27]. The module substitution strategy is increasingly recognized as an effective approach that directly targets specific functional modules within peptide sequences, thereby enhancing the functional properties and characteristics of peptides [28]. Therefore, the module substitution strategy was employed in this study to develop an effective approach for enhancing the activity of bioactive peptides.

**Figure 3:**
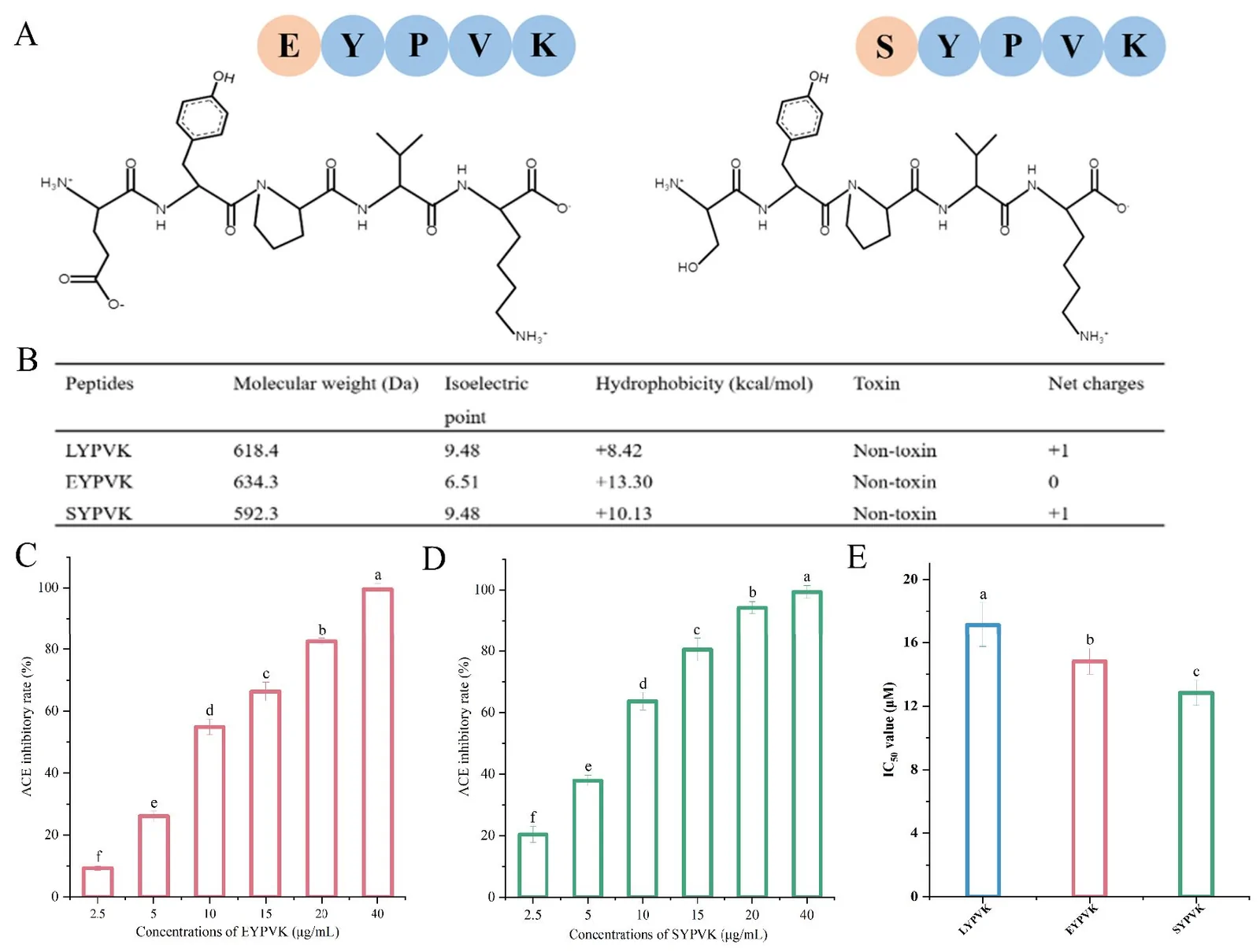
Sequence and structure of EYPVK and SYPVK obtained by substitution of modules and their ACE inhibitory activity. Sequence and structure of EYPVK and SYPVK obtained by substitution of modules (**A**). Physicochemical properties ana toxin prediction of ACE inhibitory peptides after module substitution (**B**). ACE inhibitory rates of EYPVK (**C**) and SYPVK (**D**) at their different concentrations, and IC_50_ values of the peptides against ACE (**E**). Data were presented as mean ± SD (*n* = 3). ^a–f^ Significant differences among all different peptide samples at the same time were obtained using one-way ANOVA and indicated with different letters (*p* < 0.05).

To determine whether the candidate modules identified through module-level screening could improve the activity of ACE inhibitory peptide, the N-terminal LY module of LYPVK was replaced with EY and SY, generating the full-length peptides EYPVK and SYPVK, respectively (Figure 3A). The physicochemical properties of the three peptides were further understood using in silico analysis, including molecular weight, isoelectric point, toxin, and hydrophobicity (Figure 3B). They consist of five amino acid residues with a molecular weight of 592.3–634.3 Da. The hydrophobicity of these peptides was found to be higher than that of the parent peptide LYPVK, with values ranging from +8.42 kcal/mol to +13.30 kcal/mol (Figure 3B). Additionally, the potential toxicity of the peptides was predicted using ToxinPred, and the analysis predicted all peptides to be non-toxic, which yields preliminary in silico evidence supporting the potential safety of the candidate peptides.

Meanwhile, the in vitro activity against ACE was evaluated to assess the effectiveness of the module substitution strategy employed. It was observed that the activity of EYPVK and SYPVK was enhanced to varying degrees, and their inhibitory activity also increased compared to that of LYPVK. SYPVK demonstrated the highest inhibitory potency, with an IC_50_ value of 12.83 ± 0.79 μM, followed by EYPVK, which exhibited an IC_50_ value of 14.82 ± 0.82 μM (Figure 3C–E). The ACE inhibitory peptide a with lower IC_50_ value indicates a higher level of inhibitory activity of them against ACE. The decreased IC_50_ values compared to that of LYPVK indicated that the substitution of LY with SY and EY enhanced the in vitro ACE inhibitory activity by 25.1% and 13.5%, respectively (Figure 3E). Notably, both peptides generated through the module substitution strategy exhibited enhanced ACE inhibitory activity relative to the parent peptide, supporting the feasibility of using the module substitution strategy for ACE inhibitory peptide optimization. This observation is also consistent with previous studies in which module substitution was successfully employed to improve the activities of bioactive peptides [8,9]. These findings collectively suggest that this approach has potential as an efficient rational strategy for peptide modification. Nevertheless, the current study is limited to a single parent peptide, and the efficacy of the proposed strategy was only validated in the LYPVK. Future studies should systematically evaluate peptides with diverse sequences, lengths, physicochemical properties, and characteristics to determine the feasibility of the strategy.

### 3.4. Ace Inhibitory Pattern Analysis of Designed Peptides

**Figure 4:**
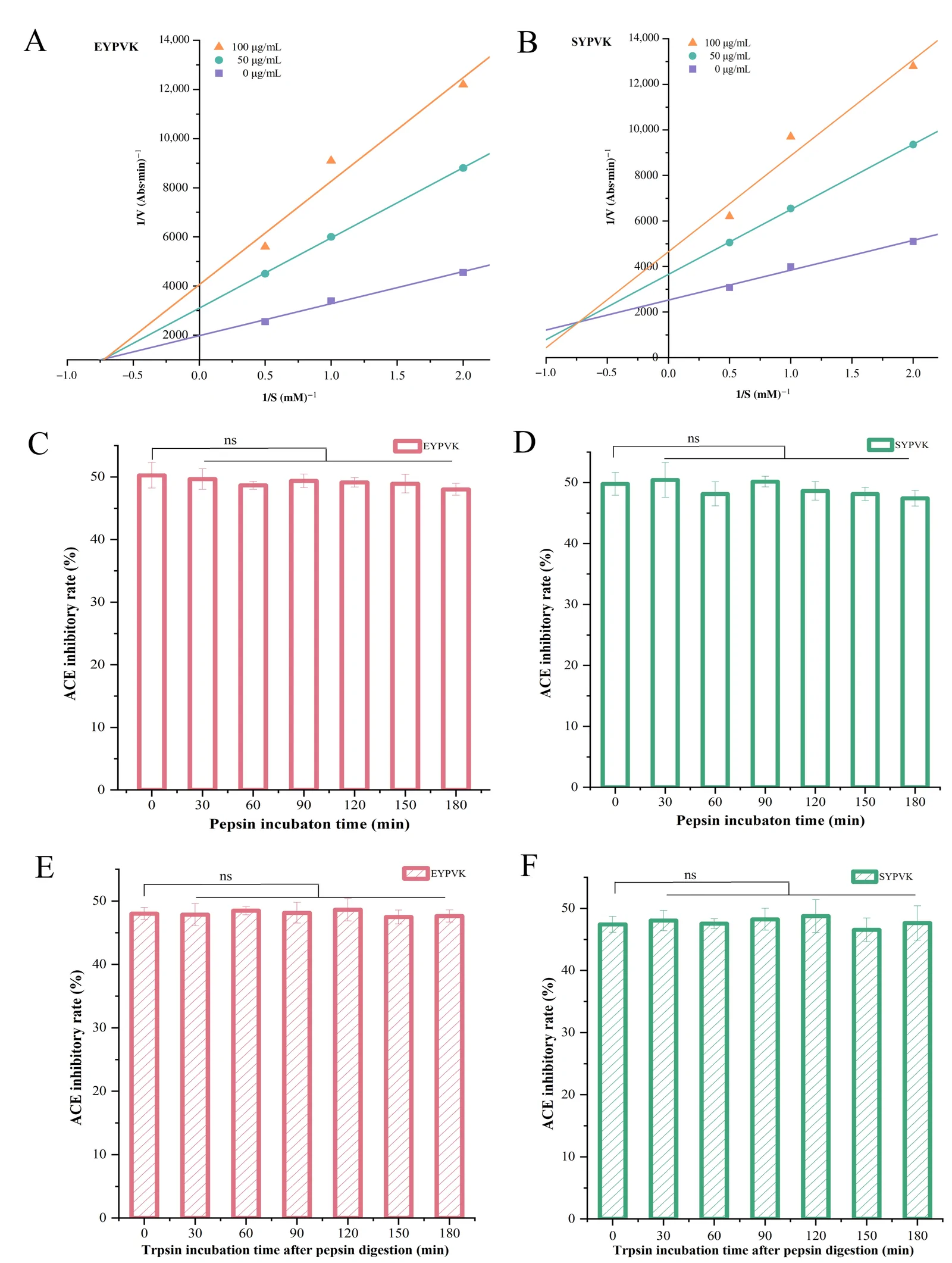
ACE inhibitory properties analysis of EYPVK and SYPVK. Determination of the ACE inhibitory pattern of EYPVK (**A**) and SYPVK (**B**). The Lineweaver–Burk plot analysis of ACE inhibition action mode of peptides was performed in the absence or presence of different peptide concentrations. Remained ACE inhibitory activity of EYPVK (**C**) and SYPVK (**D**) after in vitro simulated gastric juices digestion. Remained ACE inhibitory activity of EYPVK (**E**) and SYPVK (**F**) after the subsequently in vitro simulated intestinal fluid digestion. Data were presented as mean ± SD (*n* = 3), and ns means there is no significant differences among groups (*p* > 0.05).

Investigation of enzyme kinetics plays a crucial role in the development of ACE inhibitory peptides, as it enhances the understanding of binding interactions between ACE and substrates [10]. The Lineweaver–Burk plots were employed to examine the action mode of EYPVK and SYPVK to provide the effect of substituted modules on their inhibition mechanism. Generally, inhibition patterns of peptides mainly involve three types of modes, including competitive, noncompetitive, and mixed inhibitory mechanisms [29]. The fitted $K_{m}$ values were 0.68 mM (95% CI: 0.56–0.81 mM) and 0.82 mM (95% CI: 0.68–0.98 mM), respectively. The corresponding $V_{max}$ values were $5.10\times 10^{-4}$ (95% CI: $4.62–5.61\times 10^{-4}$) and $5.32\times 10^{-4}$ (95% CI: $4.78–5.91\times 10^{-4}$) ΔAbs min^−1^ (Tables S2 and S3). According to the fitting results, it was observed that as the concentration of EYPVK increased, K_m_ for the reaction maintained a constant trend, while its V_max_ decreased, suggesting the noncompetitive inhibition pattern of EYPVK (Figure 4A). In contrast, as the concentration of SYPVK increased, both the K_m_ and V_max_ values for the reaction decreased, suggesting that SYPVK acts as a mixed-type inhibitor (Figure 4B). This observation indicates its ability to interact with the active site and allosteric site of ACE [30].

### 3.5. In Vitro Simulated Digestion Stability Analysis of Designed Peptides

The ACE inhibitory peptide stability during in vitro digestion plays a crucial role in determining their in vivo antihypertensive effects after oral administration. During simulated gastric fluid digestion, no significant decrease in the ACE inhibitory activity was observed between the peptides with substituted modules and the parent peptide (Figure 4C,D). Specifically, for the parent peptide LYPVK, a previous study reported an ACE inhibition rate of 50.59 ± 1.64% before digestion and 48.50 ± 3.68% after 120 min of simulated gastric digestion [16]. After 120 min of simulated gastric digestion, the ACE inhibition rates measured for EYPVK and SYPVK were 48.02 ± 0.94% and 47.42 ± 1.26%, respectively (Figure 4C,D). Following 180 min of simulated intestinal digestion, the corresponding inhibition rates were 47.64 ± 0.96% and 46.54 ± 1.90%, respectively (Figure 4E,F). These results show that ACE inhibitory activity was retained under the tested digestion conditions. Nevertheless, it is worth noting that a substantial proportion of the peptide may have been cleaved by digestive enzymes, yet the residual concentration remained sufficient to maintain an inhibitory effect close to 50%. The retention of ACE inhibitory activity during simulated digestion is closely influenced by the composition and types of amino acids. Generally, Pro-containing peptide sequences exhibit remarkable resistance to simulated in vitro digestion [31,32]. This resistance may contribute to the enhanced stability of EYPVK and SYPVK, as the substituted modules do not affect the presence of proline residues in their sequences. Additionally, the small molecular weight of these peptides contributed to their favorable stability, although their bioavailability requires further investigation [33].

### 3.6. Potential Module Effect and Mechanism of Module Substitution Enhancing Peptide Activity Revealed by Molecular Docking Analysis

The aforementioned activity verification has demonstrated that module substitution is a highly effective strategy for the precise design and modification of LYPVK peptide. To elucidate the underlying molecular mechanism and potential module effect, molecular docking was conducted to analyze the interactions between peptides and ACE and the action sites before and after module substitution. The composition of catalytic center of enzyme is crucial for investigating the potential binding mechanisms. Within the ACE catalytic center, there are three active pockets, including S1, S1′, and S2, each of which contains specific key residues. The S1 pocket mainly consists of Ala354, Glu384, and Tyr523, the S2 pocket is composed of five residues including Gln281, His353, Lys511, His513, and Tyr520, and Glu162 is located in the S1′ pocket of ACE [34]. Additionally, Zn^2+^ plays a critical role in ACE inhibitory activity by binding to His383, His387, and Glu411 residues located within the active site of ACE, thereby forming a tetrahedral coordination structure [11].

**Figure 5:**
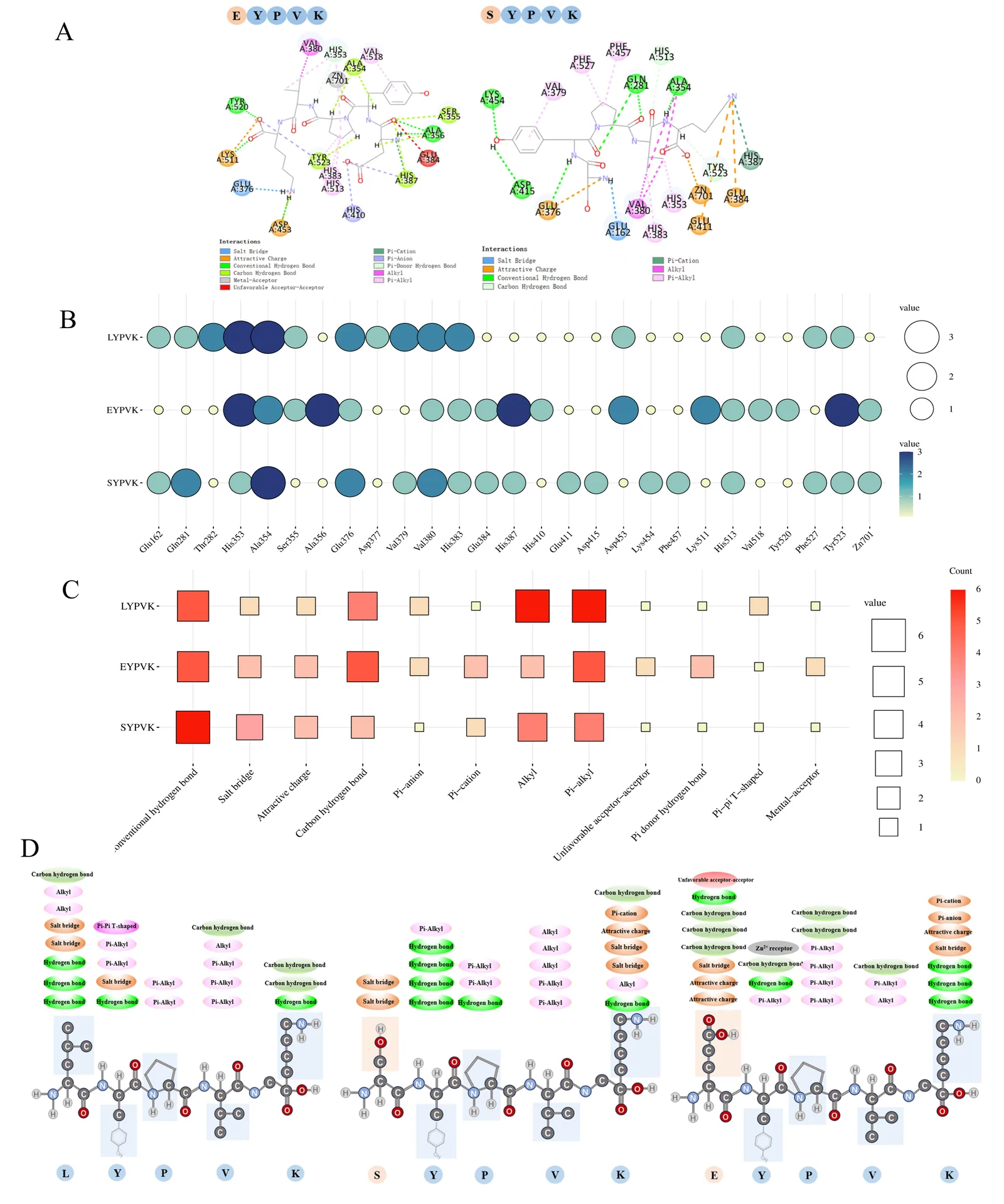
Molecular docking analysis between EYPVK and SYPVK obtained by substitution of modules and ACE. (**A**): 2D detailed diagram of the interactions between these peptides and ACE. (**B**): Interaction residue diagrams between these peptides and ACE. (**C**): Interaction type diagrams between these peptides and ACE. (**D**): Interaction profiles formed by each residue within peptide sequences.

The optimal docking conformations of EYPVK and SYPVK revealed a diverse array of non-covalent interactions formed with ACE, including electrostatic interactions, conventional and carbon–hydrogen bonds, pi–cation and pi–anion interactions, pi–pi T-shaped stacking, alkyl and pi–alkyl interactions, as well as metal–acceptor interactions. Notably, both EYPVK and SYPVK exhibited a greater variety and number of interactions compared to LYPVK. Hydrogen bonding and hydrophobic interactions are widely recognized as the primary forces driving the affinity between peptides and ACE [31]. It revealed that EYPVK and SYPVK formed a greater number of hydrogen bonds and hydrophobic interactions with ACE compared to LYPVK (Figure 5A,B). These enhanced molecular interactions are likely to contribute to increased stability of the peptide–enzyme complex and thereby may contribute to greater inhibitory potency. Specifically, the EY and SY modules introduce additional functional groups such as the polar side chain of Ser residue, which are capable of forming novel or stronger interactions with key residues within the active site. From the perspective of interaction coverage, the number of ACE residues involved in binding is positively correlated with inhibitory strength [33]. As shown in Figure 5C, the module-substituted peptides interact with a greater number of residues, which was reflected in the results showing that SYPVK engages 18 residues and EYPVK interacts with 17, whereas LYPVK contacts only 15. This expansion of the binding interface indicates the potential enhanced spatial complementarity between the substituted modules and the ACE binding pockets.

Focusing on the potential module effects, substitution of Leu in the LY module, a nonpolar aliphatic residue, with Glu or Ser introduces side chains with distinct physicochemical properties. Glu possesses an acidic carboxylate-containing side chain that is predominantly negatively charged under physiological conditions, whereas Ser contains a polar hydroxyl group capable of participating in hydrogen bonding interactions. Consistent with these chemical differences, molecular docking predicted altered interaction patterns for the substituted peptides, including salt bridge, attractive charge, conventional hydrogen-bond, carbon–hydrogen-bond, and unfavorable acceptor–acceptor interactions (Figure 5D). These predicted interaction differences provide possible structural explanations for the altered peptide–ACE interaction patterns following module substitution. Compared with the interactions of the Leu residue within the LY module of the original peptide LYPVK, the substituted Glu residue within the EY module formed a greater number of interactions. Additionally, this modification may enhance the capacity of peptide to simultaneously engage and occupy multiple pockets and thereby may contribute to improving its inhibitory activity against ACE. However, it is noteworthy that although the substitution of the Ser residue within the SY module introduces polar hydroxyl groups capable of forming specific salt bridges with active-site residues, such as Glu162 and Glu376 (Figure 5D), which may contribute to stabilizing the peptide within the catalytic groove, the Ser residue formed only two such interactions, fewer than those formed by the Leu residue in the LY module of the original peptide LYPVK. The enhanced activity of SYPVK can be attributed not only to the overall improvement in interactions but also to the presence of a potential module effect within the module. Specifically, the associated amino acids within the ACE inhibitory peptide module may exert synergistic enhancement or functional weakening effects, and peptides formed from different combinations of these modules demonstrate varying degrees of functional activity. For example, substituting the N-terminal Leu residue with either Glu or Ser not only modified the polarity and charge of the N-terminal module but also significantly affected the interaction capability of the adjacent Tyr residue (Figure 5D). Substitution with Glu or Ser may facilitate the formation of additional non-covalent interactions with amino acid residues of ACE, which has the potential to enhance the interactions between the phenolic hydroxyl group on the Tyr residue and amino acid residues within ACE active pockets. This facilitates more effective participation of Tyr residue in EYPVK and SYPVK in conventional hydrogen bonding and Zn^2+^ coordination, thereby enhancing the binding affinity of these peptides with ACE (Figure 5D).

In conclusion, the substitution of the module not only introduces new interaction sites via residues Glu or Ser but also triggers a favorable structural rearrangement of the entire peptide backbone, thereby enhancing the interaction potential of the Tyr residues. This results in enhanced hydrogen bonding, more extensive hydrophobic interactions, and improved binding occupancy within the ACE active site. These findings provide a potential mechanistic explanation for the enhanced ACE inhibitory activity experimentally observed for EYPVK and SYPVK and further suggest the presence of a potential module effect within ACE inhibitory peptides.

### 3.7. MD Simulation Analysis Revealed the Stability of Peptide–ACE Complexes

**Figure 6:**
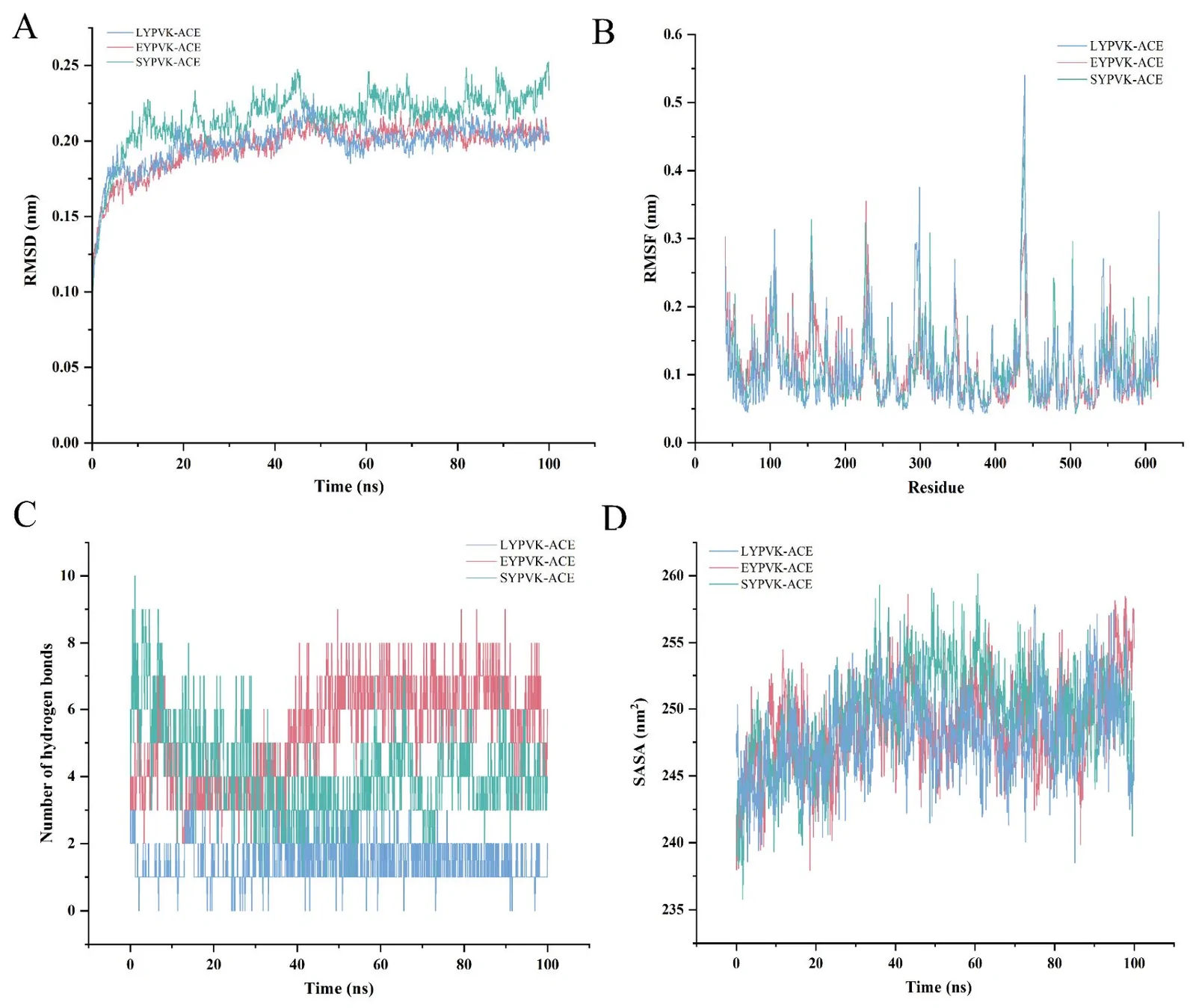
MD simulation results between LYPVK and its designed peptides after module substitution and ACE. MD simulation results conclude the variation in RMSD (**A**), RMSF (**B**), the number of hydrogen bonds (**C**), and SASA (**D**) of the complexes in the MD simulation process.

MD simulation is a computational method used to predict the structure and dynamics of substances within molecular systems, which was employed to assess the stability and identify the binding sites of peptide–ACE complexes before and after module substitution. The structural behavior of the peptide–ACE complexes during the 100 ns MD simulations was evaluated using RMSD, RMSF, hydrogen-bond, and SASA analyses. RMSD values were calculated as a measurement for the structural stability of peptide–ACE complexes. Previous studies have indicated that stronger binding affinity of ligands is associated with a potential higher RMSD value in the ligand–ACE complex [35]. After equilibration, the RMSD values of both complexes fluctuated within a relatively stable range over the simulated period (Figure 6A). It was observed that the mean RMSD values were 0.196 nm for the EYPVK–ACE complex and 0.197 nm for the SYPVK–ACE complex, indicating the comparable overall structural deviations during the simulations according to RMSD values. Additionally, RMSF is utilized to analyze the fluctuations in atomic positions of each residue throughout the MD simulation. Generally, greater RMSF values indicate higher flexibility of the amino acid residues, which may contribute to reduced binding stability. It was observed that the RMSF range of the LYPVK–ACE complex was greater than that of the SYPVK–ACE and EYPVK–ACE complexes (Figure 6B), suggesting enhanced structural stability in the complexes formed between peptides containing substituted modules and ACE.

Hydrogen bonds were subsequently analyzed because it is the primary intermolecular force that binds receptors to ligands [36]. It was observed that the most numbers of hydrogen bonds were generated between SYPVK–ACE and EYPVK–ACE complexes during the simulation process, which suggests its most stable binding interactions compared with the LYPVK–ACE complex (Figure 6C). Additionally, SASA analysis was performed to characterize the potential solvent exposure of the peptide–ACE complexes during the MD simulations. The average SASA values of the SYPVK–ACE and EYPVK–ACE complexes (249.75 ± 3.70 nm^2^) were higher than that of the SYPVK–ACE complex (248.80 ± 3.39 nm^2^) (Figure 6D), indicating differences in the overall solvent-accessible surface among these complexes. These variations may reflect differences in the conformational organization and solvent exposure of the peptide–ACE systems during the simulations. However, SASA does not directly represent peptide entry into the ACE catalytic cavity, active-site occupancy, or binding affinity, which may be used to understand the stability of the complexes during simulation. Overall, these results indicate that SYPVK and EYPVK demonstrate higher ACE inhibitory activity than LYPVK, thereby highlighting the effectiveness of the module substitution strategy in enhancing the inhibitory activity of the peptide.

### 3.8. Evaluation of Cellular Function of Peptides

**Figure 7:**
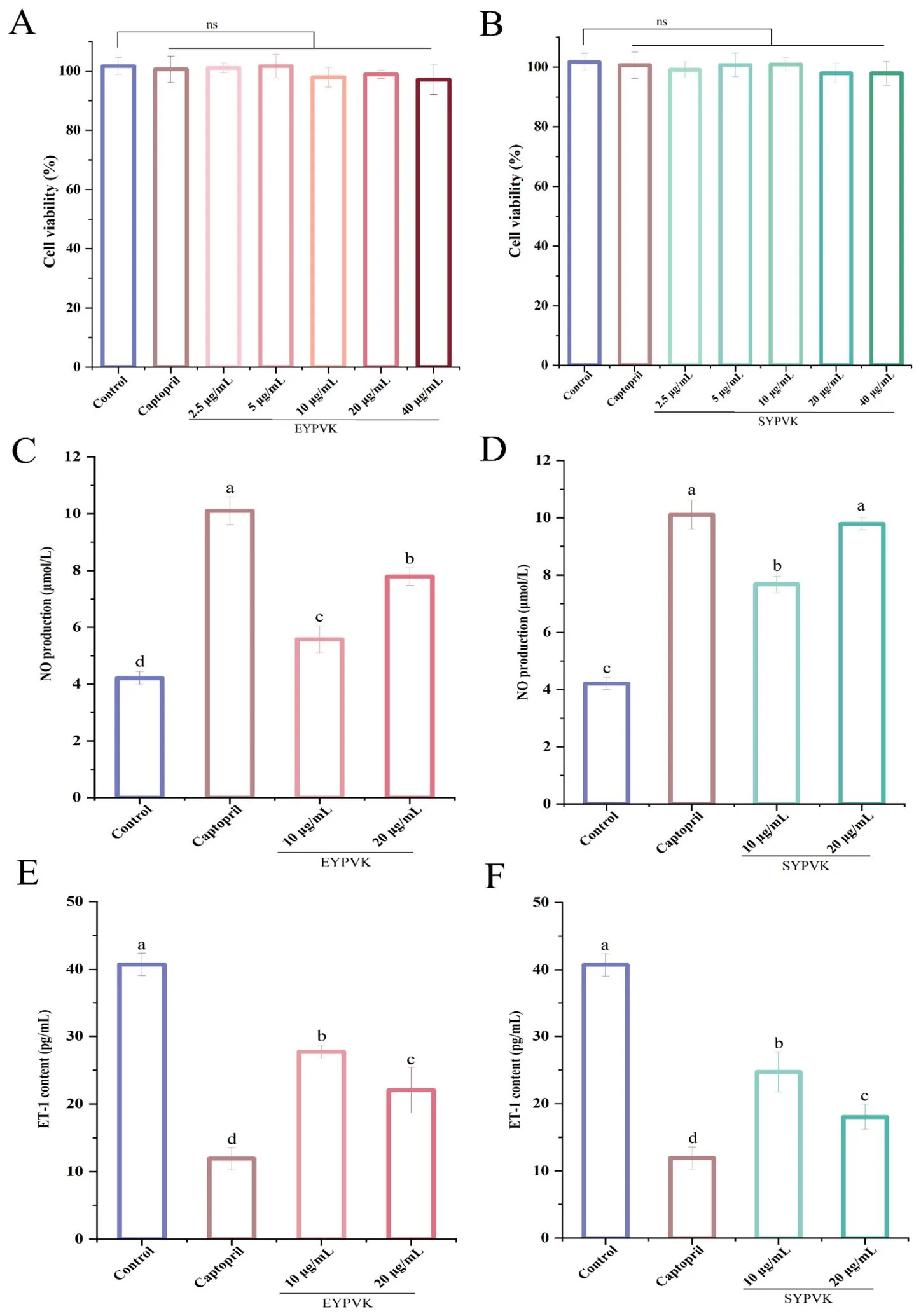
The antihypertensive effects on endothelial cells of EYPVK and SYPVK. Cytotoxicity of EYPVK (**A**) and SYPVK (**B**) at their different concentrations on EA.Hy926 cells. Effects of different concentrations of EYPVK (**C**) and SYPVK (**D**) on the release of NO. Effects of different concentrations of EYPVK (**E**) and SYPVK (**F**) on the content of ET-1. Data were presented as mean ± SD (*n* = 3). ^a–d^ Significant differences among all different peptide samples at the same time were obtained using one-way ANOVA and indicated with different letters (*p* < 0.05), and ns means there is no significant differences among groups (*p* > 0.05).

Endothelial cells, which line the interior surface of blood vessels, play a critical role in maintaining vascular homeostasis through the production of mediators that regulate vascular tone. This functional characteristic makes them a suitable in vitro model for investigating the intracellular mechanisms of peptides [34]. In this study, EA.hy926 cells were employed to assess the antihypertensive potential of peptides. Cell viability was evaluated using the CCK-8 assay at various peptide concentrations, with captopril utilized as the positive control. The results demonstrated there was no apparent cytotoxicity under the tested experimental conditions, as cell viability exceeded 90% across all peptide-treated groups (Figure 7A,B).

NO, a key endothelial-derived factor, plays a direct role in lowering blood pressure through the mitigation of angiotensin II effects and the suppression of ACE expression. EA.hy926 cells were treated with 10 and 20 μg/mL of each peptide for 24 h to assess the NO release levels. It was demonstrated that all peptide-treated groups exhibited significantly increased NO production compared to the control group, suggesting their potential function at the cellular levels (Figure 7C,D). Meanwhile, a dose-dependent increase in NO production was observed, aligning with the trends of other reported ACE inhibitory peptides [32,34]. Importantly, the peptides modified through module substitution elicited higher NO production compared to the original peptide LYPVK, with EYPVK and SYPVK demonstrating notably enhanced effects.

Additionally, many ACE inhibitors, including captopril and reported peptides like IVTNWDDMEK and SFYPDY, have demonstrated the capacity to suppress the ET-1 secretion in endothelial cells [34]. In this study, the levels of ET-1 in the culture medium of EA.hy926 were markedly decreased following the treatment with the tested peptides (Figure 7E,F). Notably, peptides modified through module substitution demonstrated a more significant reduction in ET-1 levels compared to LYPVK, further supporting their enhanced bioactivity. These findings align with the observed enhancements in ACE inhibitory activity, thereby supporting the efficacy of module substitution in optimizing the antihypertensive properties of peptide.

### 3.9. Potential Antihypertension Mechanism Mining for SYPVK by Network Pharmacology Analysis

**Figure 8:**
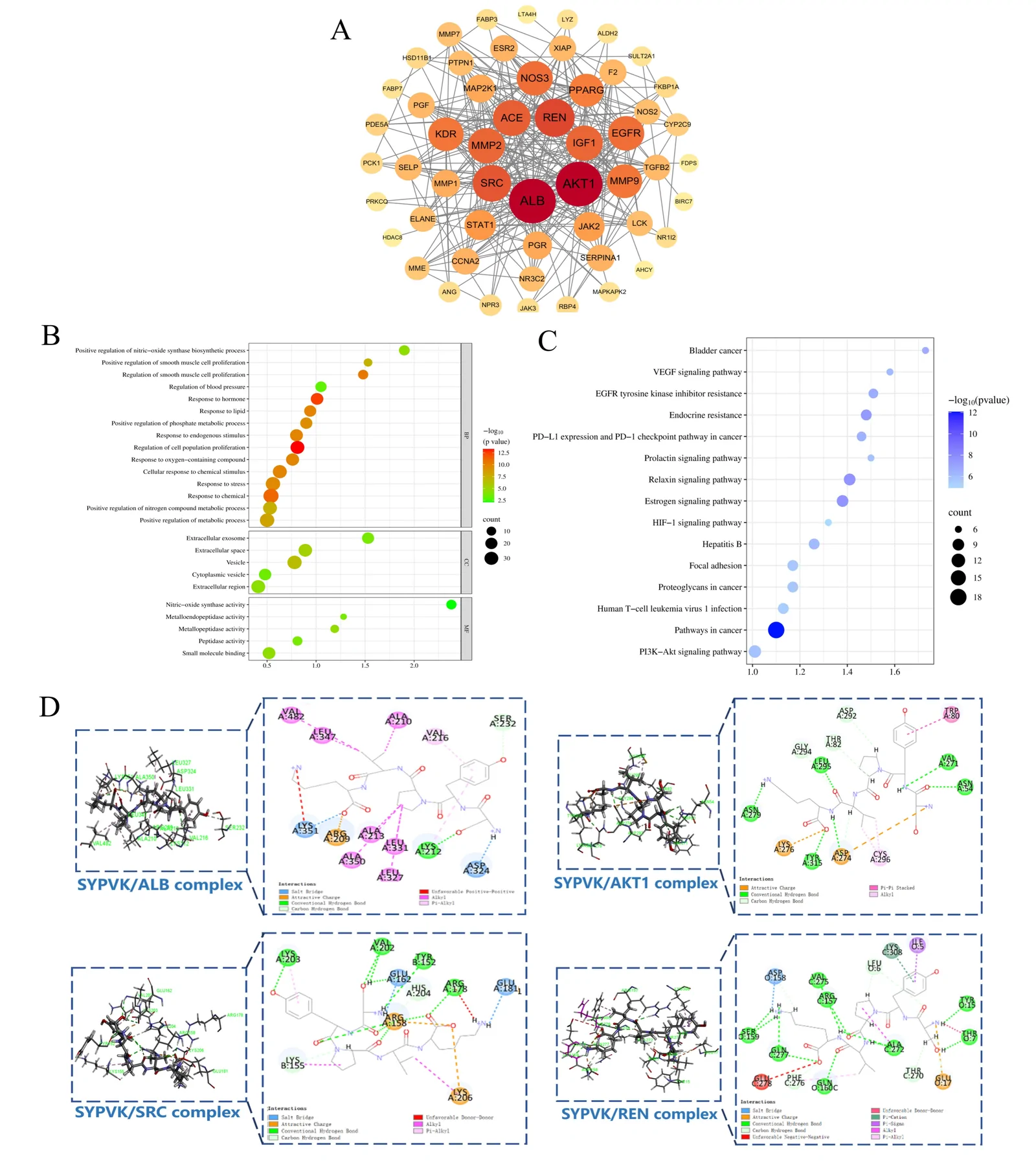
The potential antihypertensive mechanism mining of SYPVK. (**A**): The PPI network of the potential hypertension-related targets of SYPVK. (**B**): GO enrichment analysis of potential hypertension-related targets of SYPVK using the bubble diagram. The size of each bubble corresponded with gene count in a particular pathway, and the enrichment significance was shown by the color saturation of the bubble. (**C**): KEGG enrichment analysis of potential targets of SYPVK using the bubble diagram. The size of each bubble corresponded to gene expressions in a particular pathway, and the enrichment significance was shown by the color saturation of the bubble. (**D**): Docking interactions of SYPVK with the potential targets, including AKT1, REN, ALB, and SRC.

Network pharmacology is extensively utilized to predict potential drug targets for specific diseases through computational-aided analysis. The pathogenesis of hypertension is a complex process that may involve multiple interconnected molecular targets. To further explore the potential targets of SYPVK resulting from module substitution, a network pharmacology approach was applied. A total of 55 target sets of SYPVK were identified and compiled, forming a comprehensive set of candidate targets of SYPVK (Table S3). PPI network analysis was conducted using the STRING database based on the identified target set and visualized with Cytoscape software to identify potential key targets of SYPVK. As the degree value of a target within the PPI network increases, the importance and centrality of that target within the network also increase, thereby indicating that AKT1, ALB, SRC, ACE, and REN are the primary potential targets (Figure 8A). These genes encode proteins that play essential roles in a wide array of cellular functions that are related to hypertension. Specifically, AKT1 mediates key biological processes (BP) such as metabolism, cell growth, and apoptosis. It is also involved in the phosphorylation of eNOS, angiogenesis, and vasorelaxation [37]. The serum protein ALB is involved in the regulation of various physiological processes, including blood flow, blood pressure, and angiogenesis [38]. The non-receptor tyrosine kinase SRC plays a pivotal role in maintaining normal physiological functions, including its involvement in the initiation, progression, migration, and invasion of tumors [39]. REN regulates blood pressure through activating the activity of key substances presented in the RAS system [40].

The most significant GO terms were visually presented in the enrichment analysis diagram, including BP, molecular functions (MF), and cellular components (CC) (Figure 8B). Specifically, BP was predominantly involved in positive regulation of nitric oxide synthase biosynthetic process, positive regulation of smooth muscle cell proliferation, regulation of blood pressure, and response to hormone. CC was mainly enriched in extracellular exosome, vesicle, and extracellular space. MF was mainly enriched in nitric oxide synthase activity and metalloendopeptidase activity. Meanwhile, KEGG enrichment analysis identified a diverse array of associated signaling pathways, with the top three being pathways in bladder cancer, pathways in cancer, and VEGF signaling pathway (Figure 8C). Despite the highly ranked pathways not being directly associated with hypertension, the predicted target set contains broadly connected signaling proteins that has potential effects on hypertension regulation. In this regard, it was suggested that SYPVK may exert its antihypertensive effect through the modulation of multiple targets and the regulation of various pathways.

The molecular docking analysis was further conducted to investigate the binding activity and interactions between the SYPVK and core potential hypertensive targets. The four targets—AKT1, ALB, SRC, and REN—exhibiting the highest degree values in the PPI network, apart from ACE, were subjected to docking analysis with the SYPVK. The 2D and 3D interaction forms between the SYPVK, and the key targets revealed that the conventional hydrogen bond is the main force contributing to their binding, followed by the salt bridge, attractive charge, and the alkyl and pi–alkyl interaction forces (Figure 8D). The successful molecular docking between the predicted key targets and SYPVK validates the potential of utilizing a network pharmacological approach to identify the promising action targets.

### 3.10. Effect of SYPVK on Key Targets Gene Expression in EA.Hy926 Cells

**Figure 9:**
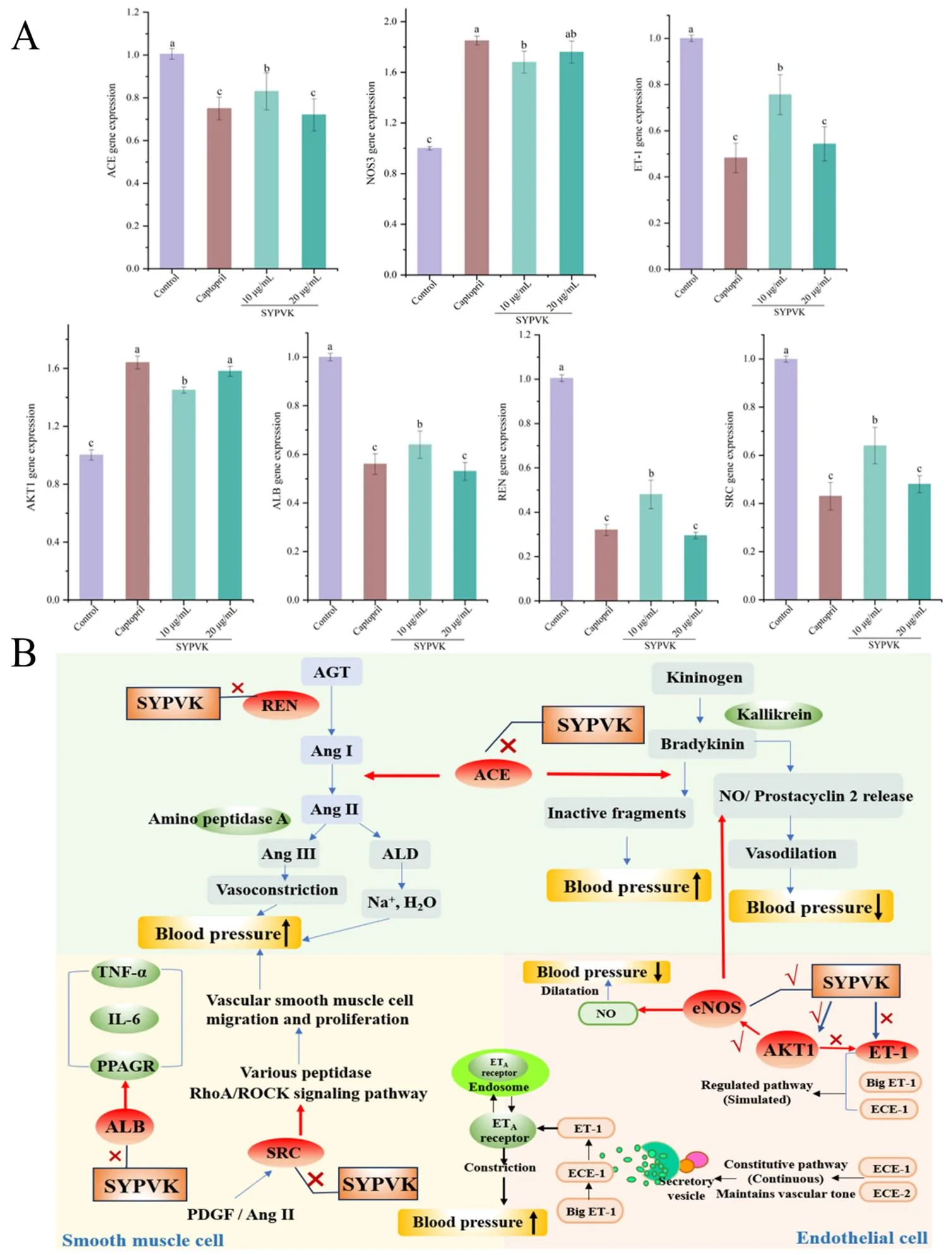
(**A**): Effects of SYPVK on the relative gene expression of potential targets in EA.Hy926 cells. (**B**): Overall diagram of the potential antihypertensive action mechanism of SYPVK. Data were presented as mean ± SD (*n* = 3). ^a–c^ Significant differences among all different peptide samples at the same time were obtained using one-way ANOVA and indicated with different letters (*p* < 0.05).

The effects of SYPVK on the potential key genes expression related to the blood pressure regulation system were measured to investigate its potential mechanism. The results demonstrated that the expression of *ACE* mRNA in endothelial cells treated with different concentrations (10 and 20 µg/mL) of SYPVK was significantly suppressed (Figure 9A). It has been reported that the phosphorylation of endothelial eNOS accounts for the increased production of NO, and the expression of ET-1 is associated with the secretory content of ET-1, which contributes to the improvement of endothelial function [34]. It was observed that treatment with SYPVK significantly promoted the gene expression of *NOS3* in a dose-dependent manner while reducing the expression of *ET-1* (Figure 9A). Additionally, the gene expression of *REN*, *AKT1*, *ALB*, and *SRC* was also altered by SYPVK, suggesting that the peptide has the potential to exert its functions by affecting the key gene expression.

The aforementioned findings indicate that SYPVK might exert antihypertensive effects by modulating potential hypertension-associated targets, such as *NOS3*, *ACE*, *REN*, *ALB*, *AKT1*, and *SRC*. Based on the relevant pathways within the blood pressure system and the up-regulation or down-regulation of the gene expression of key potential targets induced by SYPVK, the potential antihypertensive action mechanism is presented in Figure 9B. Specifically, SYPVK may exert its antihypertensive effect by binding to ACE and REN, thereby inhibiting their activities and influencing the RAS and KKS systems associated with hypertension. SYPVK may also interact with eNOS to activate the enzymatic activities and metabolic processes, thereby promoting the release of NO. Based on the decreased ET-1 content in endothelial cells and the change in its gene expression, SYPVK also has the potential to interact with the ET-1 to exert an antihypertensive effect. Despite the fact that ALB, SRC, and AKT1 do not directly affect the blood pressure level, these substances are known to be associated with the inflammatory response, angiogenesis, and vasodilation. It is likely that SYPVK inhibits these factors, thereby facilitating the migration and proliferation of vascular smooth muscle cells, as well as vasodilation, to exert its antihypertensive effect. Overall, SYPVK may exert antihypertensive effects via multiple targets and pathways.

## 4. Conclusions

We demonstrated that rational dipeptide module substitution is an effective strategy for enhancing the ACE inhibitory activity of bioactive peptides in this study. By integrating molecular docking with in vitro activity assays, the key functional modules within the peptide LYPVK were identified, including the low-activity module LY and the higher module YP. Guided by these insights, the low-contributing dipeptide module LY was replaced with two high-performance modules, EY and SY, leading to the design of the peptides EYPVK and SYPVK. These modified sequences exhibited significantly improved ACE inhibitory activity, with increases of 13.5% and 25.1%, respectively, compared to the parent peptide. Molecular docking analysis revealed that the enhanced activity could be attributed to stronger hydrogen bonding and hydrophobic interactions with the ACE active site. Meanwhile, it was demonstrated that the potential module effect, wherein the Glu or Ser residues within substituted modules influenced the interactions of the associated Tyr residue with ACE, enhanced the binding affinity of the peptide with ACE. Furthermore, functional assays in vascular endothelial cells demonstrated that the substituted peptides promoted their functions by increasing NO release and reducing ET-1 levels. Additionally, the biological functions of SYPVK may potentially regulate blood pressure through interactions with key regulatory targets, which broadens its therapeutic potential. Overall, this study provides a rational approach for the development of functional peptides with enhanced bioactivity while offering innovative insights into the potential module effect within ACE inhibitory peptide sequence.

## Data Availability

The original contributions presented in this study are included in the article/Supplementary Materials. Further inquiries can be directed to the corresponding authors.

## Supplementary Materials

The following supporting information can be downloaded at: https://www.mdpi.com/article/10.3390/foods15203617/s1, Figure S1. Interactions between dipeptide SY and EY binding to ACE, respectively; Table S1. Primer sequences used for RT-qPCR analysis; Table S2. Comparison of inhibition models for ACE inhibition; Table S3. Kinetic parameters obtained from nonlinear global fitting; Table S4. PPI analysis of potential targets of SYPVK.

## References

[1] Kario K., Okura A., Hoshide S., Mogi M. (2024). The WHO global report 2023 on hypertension warning the emerging hypertension burden in globe and its treatment strategy. Hypertens. Res..

[2] Bryniarski P., Nazimek K., Marcinkiewicz J. (2022). Immunomodulatory activity of the most commonly used antihypertensive drugs-angiotensin converting enzyme inhibitors and angiotensin II receptor blockers. Int. J. Mol. Sci..

[3] Abdo A.A., Al-Dalali S., Hou Y., Aleryani H., Shehzad Q., Asawmahi O., AL-Farga A., Mohammed B., Liu X., Sang Y. (2024). Modification of marine bioactive peptides: Strategy to improve the biological activity, stability, and taste properties. Food Bioprocess Technol..

[4] Ding Q., Sheikh A.R., Chen Q., Hu Y., Sun N., Su X., Luo L., Ma H.L., He R. (2023). Understanding the mechanism for the structure-activity relationship of food derived ACEI peptides. Food Rev. Int..

[5] Xue L., Yin R., Howell K., Zhang P. (2021). Activity and bioavailability of food protein derived angiotensin-I-converting enzyme–inhibitory peptides. Compr. Rev. Food Sci. Food Saf..

[6] Fan H., Liao W., Wu J. (2019). Molecular interactions, bioavailability, and cellular mechanisms of angiotensin-converting enzyme inhibitory peptides. J. Food Biochem..

[7] Xiang L., Qiu Z., Zhao R., Zheng Z., Qiao X. (2023). Advancement and prospects of production, transport, functional activity and structure-activity relationship of food-derived angiotensin converting enzyme (ACE) inhibitory peptides. Crit. Rev. Food Sci..

[8] Meng P., Wang Y., Huang Y., Liu T., Ma M., Han J., Su X., Li W., Wang Y., Lu C. (2024). A strategy to boost xanthine oxidase and angiotensin converting enzyme inhibitory activities of peptides via molecular docking and module substitution. Food Chem..

[9] Su L., Ma Z., Ji H., Kong J., Yan W., Zhang Q., Li J., Zuo M. (2025). From prediction to design: Revealing the mechanisms of umami peptides using interpretable deep learning, quantum chemical simulations, and module substitution. Food Chem..

[10] Zhang L., Miao J., Guo J., Liu J., Xia Z., Chen B., Ma F., Cao Y. (2023). Two novel angiotensin I-converting enzyme (ACE) inhibitory peptides from rice (*Oryza sativa* L.) bran protein. J. Agric. Food Chem..

[11] Xu Z., Wu C., Sun-Waterhouse D., Zhao T., Waterhouse G.I., Zhao M., Su G. (2021). Identification of post-digestion angiotensin-I converting enzyme (ACE) inhibitory peptides from soybean protein isolate: Their production conditions and in silico molecular docking with ACE. Food Chem..

[12] Liang Q., Liu Z., Xiao M., Zhou W., Yu D., Liu J., Wang L., Mou H. (2025). Improving activity of ACE inhibitory peptide by sequence-based rational design: Properties, function on endothelial cells, and potential antihypertensive mechanism. Food Front..

[13] Wang X., Shen Y., Wang S., Li S., Zhang W., Liu X., Lai L., Pei J., Li H. (2017). PharmMapper 2017 update: A web server for potential drug target identification with a comprehensive target pharmacophore database. Nucleic Acids Res..

[14] Sherman B.T., Hao M., Qiu J., Jiao X., Baseler M.W., Lane H.C., Imamichi T., Chang W. (2022). DAVID: A web server for functional enrichment analysis and functional annotation of gene lists (2021 update). Nucleic Acids Res..

[15] Cheng J., Xiao M., Ren X., Secundo F., Yu Y., Nan S., Chen W., Zhu C., Kong Q., Huang Y. (2023). Response of *salmonella enterica* serovar typhimurium to alginate oligosaccharides fermented with fecal inoculum: Integrated transcriptomic and metabolomic analyses. Mar. Life Sci. Technol..

[16] Liang Q., Liu Z., Xu M., Zhu J., Liang Z., Zhu C., Mou H. (2025). Heterologous expression of a recombinant ACE inhibitory peptide LYPVK and its potential antihypertensive action mechanism. Int. J. Biol. Macromol..

[17] Kamei Y., Hatazawa Y., Uchitomi R., Yoshimura R., Miura S. (2020). Regulation of skeletal muscle function by amino acids. Nutrients.

[18] Chen M., Ma A., Sun Z., Xie B., Shi L., Chen S., Chen L., Xiong G., Wang L., Wu W. (2023). Enhancing activity of food protein-derived peptides: An overview of pretreatment, preparation, and modification methods. Compr. Rev. Food Sci. Food Saf..

[19] Liang Q., Liu Z., Liang Z., Fu X., Li D., Zhu C., Kong Q., Mou H. (2025). Current challenges and development strategies of bacteriocins produced by lactic acid bacteria applied in the food industry. Compr. Rev. Food Sci. Food Saf..

[20] Wang Y.T., Russo D.P., Liu C., Zhou Q., Zhu H., Zhang Y.H. (2020). Predictive modeling of angiotensin I-converting enzyme inhibitory peptides using various machine learning approaches. J. Agric. Food Chem..

[21] Ramaswamy A., Balasubramanian S., Rajagopalan M., Coumar M.S. (2021). Chapter 12-Biomolecular Talks-Part 2: Applications and challenges of molecular docking approaches. Molecular Docking for Computer-Aided Drug Design.

[22] Chen J., Yu X., Chen Q., Wu Q., He Q. (2022). Screening and mechanisms of novel angiotensin-I-converting enzyme inhibitory peptides from rabbit meat proteins: A combined in silico and in vitro study. Food Chem..

[23] Zhong C., Sun L.C., Yan L.J., Lin Y.C., Liu G.M., Cao M.J. (2018). Production, optimisation and characterisation of angiotensin converting enzyme inhibitory peptides from sea cucumber (*Stichopus japonicus*) gonad. Food Funct..

[24] Han J., Huang Z., Tang S., Lu C., Wan H., Zhou J., Li Y., Ming T., Wang Z.J., Su X. (2020). The novel peptides ICRD and LCGEC screened from tuna roe show antioxidative activity *via* Keap1/Nrf2-ARE pathway regulation and gut microbiota modulation. Food Chem..

[25] Heo S.Y., Kang N., Kim E.A., Kim J., Lee S.H., Ahn G., Oh J.H., Shin A.Y., Kim D., Heo S.J. (2023). Purification and molecular docking study on the angiotensin I-converting enzyme (ACE)-inhibitory peptide isolated from hydrolysates of the deep-sea mussel *Gigantidas vrijenhoeki*. Mar. Drugs.

[26] Grisoni F., Neuhaus C.S., Hishinuma M., Gabernet G., Hiss J.A., Kotera M., Schneider G. (2019). *De novo* design of anticancer peptides by ensemble artificial neural networks. J. Mol. Model..

[27] Rhys G.G., Cross J.A., Dawson W.M., Thompson H.F., Shanmugaratnam S., Savery N.J., Dodding M.P., Höcker B., Woolfson D.N. (2022). *De novo* designed peptides for cellular delivery and subcellular localization. Nat. Chem. Biol..

[28] Cui Z., Yu Y., Zhou T., Qi C., Gu J., Zhang N., Feng X., Zhang Z., Zhu Y., Zhang Y. (2025). Cyclization: A potential effective modification strategy for umami peptides. Food Chem..

[29] Zou L., Zhou Y., Yu X., Chen C., Xiao G. (2023). Angiotensin I-converting enzyme inhibitory activity of two peptides derived from in vitro digestion products of pork sausage with partial substitution of NaCl by KCl. J. Agric. Food Chem..

[30] Wang S., Zhang L., Wang H., Hu Z., Xie X., Chen H., Tu Z. (2024). Identification of novel angiotensin converting enzyme (ACE) inhibitory peptides from Pacific saury: In vivo antihypertensive effect and transport route. Int. J. Biol. Macromol..

[31] Chen L., Wang L., Shu G., Li J. (2021). Antihypertensive potential of plant foods: Research progress and prospect of plant-derived angiotensin-converting enzyme inhibition compounds. J. Agric. Food Chem..

[32] Hao X., Yang W., Zhu Q., Zhang G., Zhang X., Liu L., Li X., Hussain M., Ni C., Jiang X. (2021). Proteolysis and ACE-inhibitory peptide profile of Cheddar cheese: Effect of digestion treatment and different probiotics. LWT-Food Sci. Technol..

[33] Yin Z., Yan R., Jiang Y., Feng S., Sun H., Sun J., Zhao D., Li H., Wang B., Zhang N. (2022). Identification of peptides in Qingke baijiu and evaluation of its angiotensin converting enzyme (ACE) inhibitory activity and stability. Food Chem..

[34] Wang C.X., Song C.C., Liu X.T., Qiao B.W., Song S., Fu Y.H. (2022). ACE inhibitory activities of two peptides derived from *Volutharpa ampullacea* perryi hydrolysate and their protective effects on H_2_O_2_ induced HUVECs injury. Food Res. Int..

[35] Zhang B., Liu J., Wen H., Jiang F., Wang E., Zhang T. (2022). Structural requirements and interaction mechanisms of ACE inhibitory peptides: Molecular simulation and thermodynamics studies on LAPYK and its modified peptides. Food Sci. Hum. Well..

[36] Zeng J., Lin K., Zhang X., Zou J., Zhang L., Gong P., Zhao J., Han C., Liu Y., Yi H. (2023). Insight into the molecular-level details of αs1 casein interactions with IgG: Combining with LC-MS/MS and molecular modelling techniques. Food Chem..

[37] Abeyrathna P., Su Y. (2015). The critical role of Akt in cardiovascular function. Vasc. Pharmacol..

[38] Merlot A.M., Kalinowski D.S., Richardson D.R. (2014). Unraveling the mysteries of serum albumin—More than just a serum protein. Front. Physiol..

[39] Roskoski R. (2015). Src protein-tyrosine kinase structure, mechanism, and small molecule inhibitors. Pharmacol. Res..

[40] Nguyen G., Delarue F., Burcklé C., Bouzhir L., Giller T., Sraer J.D. (2002). Pivotal role of the renin/prorenin receptor in angiotensin II production and cellular responses to renin. J. Clin. Investig..

