# Supplementary material for: A Dipeptide Module Substitution-Based Strategy for the Modification and Evaluation of ACE Inhibitory Peptides
Source: Foods. 2026 Oct 11;15(20):3617. doi: 10.3390/foods15203617 (PMC13704619; doi:10.3390/foods15203617)
Supplement: Supplementary file 1 [file foods-15-03617-s001.zip › foods-4592997-supplementary.pdf]

Supplementary materials

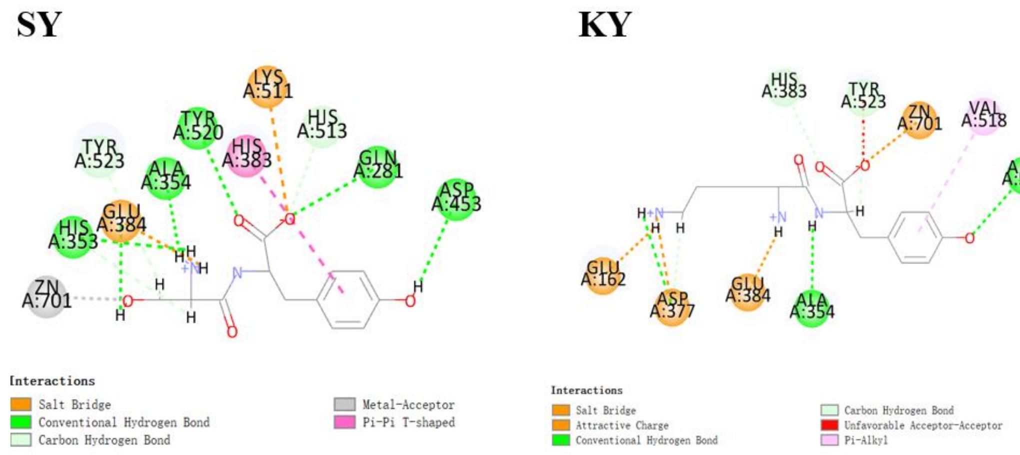

Figure S1. Interactions between dipeptide SY and EY binding to ACE, respectively.

Table S1. Primer sequences used for RT-qPCR analysis

Gene	Accession No.	Forward primer (5'–3')	Reverse primer (5'–3')
ACE	NM_000789	CATCACCACAGAGACCAGCAAG	CCGCTTGATAGTGGTGTTCTGC
AKT1	NM_005163	TGGACTACCTGCACTCGGAGAA	GTGCCGCAAAAGGTCTTCATGG
ALB	NM_000477	GATGAGATGCCTGCTGACTTGC	CACGACAGAGTAATCAGGATGCC
eNOS	NM_000603	GAAGGCGACAATCCTGTATGGC	TGTTCGAGGGACACCACGTCAT
ET-1	NM_001955	CTACTTCTGCCACCTGGACATC	TCACGGTCTGTTGCCTTTGTGG
REN	NM_000537	GAAGAGGCTGACACTTGGCAAC	GCACCCAAACATTGGACGAACC
SRC	NM_005417	CTGCTTTGGCGAGGTGTGGATG	CCACAGCATACAACTGCACCAG
GAPDH	NM_002046	GTCTCCTCTGACTTCAACAGCG	ACCACCCTGTTGCTGTAGCCAA

Table S2. Comparison of inhibition models for ACE inhibition

Peptides	Inhibition mode	RSS	R^2	AICc	ΔAICc
EYPVK	Noncompetitive	0.0039	0.988	33.2	0
SYPVK	Mixed	0.0058	0.989	37.4	0

Table S3. Kinetic parameters obtained from nonlinear global fitting

Peptides	Inhibition mode	K_m (mM)	V_{max} (ΔAbs min^{-1})	Ki (μg/mL)	Ki′ (μg/mL)
EYPVK	Noncompetitive	0.68 (0.56–0.81)	5.10×10^{-4} ($4.62–5.61\times10^{-4}$)	65.4 (53.2–80.5)	= Ki
SYPVK	Mixed	0.82 (0.68–0.98)	5.32×10^{-4} ($4.78–5.91\times10^{-4}$)	45.6 (36.1–57.8)	82.3 (64.9–105.4)

Table S4. PPI analysis of potential targets of SYPVK

	Average Shortest Path Length	Clustering Coefficient	Closeness Centrality	Degree	Betweenness Centrality	Neighborhood Connectivity	Radiality	Topological Coefficient
AKT1	1.301887	0.306306	0.768116	37	0.229198	13.43243	0.991841	0.257796
ALB	1.301887	0.328829	0.768116	37	0.187295	14.02703	0.991841	0.264661
REN	1.773585	0.544118	0.56383	28	0.017345	18	0.979092	0.375
SRC	1.566038	0.476923	0.638554	26	3.89E-02	16.53846	0.984702	0.330769
ACE	1.698113	0.442105	0.588889	25	0.047627	16.8	0.981132	0.342857
IGF1	1.622642	0.532609	0.616279	24	0.030668	17.79167	0.983172	0.363095
MMP2	1.603774	0.572464	0.623529	24	1.83E-02	18.04167	0.983682	0.360833

NOS3	1.773585	0.602941	0.56383	23	0.016212	18.88235	0.979092	0.393382
EGFR	1.622642	0.561265	0.616279	23	0.028044	17.52174	0.983172	0.350435
KDR	1.679245	0.558442	0.595506	22	0.023216	17.63636	0.981642	0.367424
MMP9	1.509434	0.473545	0.6625	22	0.049875	16.53571	0.986232	0.32423
PPARG	1.641509	0.504762	0.609195	21	0.044768	17.7619	0.982662	0.348273
STAT1	1.735849	0.602941	0.576087	17	0.019839	18.76471	0.980112	0.375294
JAK2	1.792453	0.666667	0.557895	16	0.013621	19.9375	0.978582	0.415365
MAP2K1	1.867925	0.813187	0.535354	14	0.00347	21.85714	0.976543	0.475155
PGR	1.830189	0.736264	0.546392	14	0.00359	21	0.977562	0.4375
MMP1	1.849057	0.730769	0.540816	13	0.006444	21.15385	0.977053	0.440705
SERPINA1	1.924528	0.393939	0.519608	12	0.018801	15.66667	0.975013	0.348148
ESR2	1.886792	0.848485	0.53	12	0.001276	23.58333	0.976033	0.501773
CCNA2	1.886792	0.69697	0.53	12	0.00825	20.33333	0.976033	0.432624
F2	1.962264	0.436364	0.509615	11	0.008479	15.36364	0.973993	0.349174
SELP	1.924528	0.763636	0.519608	11	0.001881	22.63636	0.975013	0.492095
XIAP	1.90566	0.618182	0.524752	11	0.074907	20.18182	0.975523	0.444444
TGFB2	1.90566	0.854545	0.524752	11	1.17E-03	23.36364	0.975523	0.497099
PGF	1.90566	0.763636	0.524752	11	0.004688	22.18182	0.975523	0.471954
LCK	1.981132	0.555556	0.504762	10	0.009817	18.5	0.973483	0.420455
ELANE	1.962264	0.577778	0.509615	10	0.005693	18.5	0.973993	0.411111
PTPN1	1.924528	0.8	0.519608	10	0.003847	23	0.975013	0.489362
NR3C2	1.90566	0.466667	0.524752	10	0.015215	17.5	0.975523	0.370213
NOS2	1.886792	0.533333	0.53	10	0.040667	19.3	0.976033	0.4
MMP7	1.962264	0.916667	0.509615	9	3.88E-04	24.66667	0.973993	0.536232
MME	1.962264	0.714286	0.509615	8	4.39E-03	21	0.973993	0.446809
CYP2C9	2.075472	0.380952	0.481818	7	0.015037	12.85714	0.970933	0.306122
PDE5A	2.075472	0.8	0.481818	6	0.002038	17.66667	0.970933	0.410853
NPR3	2.490566	0.8	0.401515	5	2.85E-04	13.6	0.959714	0.544
HSD11B1	2.283019	0.2	0.438017	5	0.010314	12.6	0.965324	0.369697
FKBP1A	2.169811	0.6	0.46087	5	0.001455	18.6	0.968383	0.484211
RBP4	2.150943	0.6	0.464912	5	7.08E-04	17.6	0.968893	0.44
PCK1	2.037736	0.4	0.490741	5	0.039467	20.2	0.971953	0.444444
JAK3	2.45283	1	0.407692	4	0	16.5	0.960734	0.611111
ANG	2.396226	0.833333	0.417323	4	8.06E-05	18.5	0.962264	0.616667
FABP3	2.169811	0.166667	0.46087	4	0.00249	16.5	0.968383	0.421053
NR1I2	2.075472	0.5	0.481818	4	0.015228	21	0.970933	0.466667
SULT2A1	2.792453	0.333333	0.358108	3	0.001451	5.333333	0.951555	0.416667
ALDH2	2.45283	0.666667	0.407692	3	2.42E-04	14	0.960734	0.482759
FABP7	2.226415	0.333333	0.449153	3	8.91E-04	17.66667	0.966854	0.468468
LYZ	2.226415	1	0.449153	3	0.00E+00	19.66667	0.966854	0.517544
MAPKAPK2	2.207547	1	0.452991	3	0.00E+00	28.33333	0.967364	0.726496
PRKCQ	2.226415	1	0.449153	2	0	23.5	0.966854	0.602564
FDPS	3.018868	0	0.33125	1	0	5	0.945436	0
AHCY	2.886792	0	0.346405	1	0	11	0.949006	0
FDPS	3.018868	0	0.33125	1	0	5	0.945436	0
HSD11B1	2.283019	0.2	0.438017	5	0.010314	12.6	0.965324	0.369697
LTA4H	2.867925	0	0.348684	1	0	10	0.949516	0
